# Moderate mechanical stress suppresses chondrocyte ferroptosis in osteoarthritis by regulating NF-κB p65/GPX4 signaling pathway

**DOI:** 10.1038/s41598-024-55629-x

**Published:** 2024-03-01

**Authors:** Juanjuan Han, Li-nan Zhan, Yue Huang, Shijia Guo, Xiaoding Zhou, Leonid Kapilevich, Zhuo Wang, Ke Ning, Mingli Sun, Xin-an Zhang

**Affiliations:** 1 College of Exercise and Health, Shenyang Sport University, Shenyang, 110100 China; 2 Department of Sport Rehabilitation, Shanghai University of Sport, Shanghai, 200438 China; 3 Faculty of Physical Education, National Research Tomsk State University, Tomsk, Russia

**Keywords:** Mechanical stress, Osteoarthritis, Ferroptosis, Cartilage, Cell death, Mechanisms of disease

## Abstract

Ferroptosis is a recently identified form of programmed cell death that plays an important role in the pathophysiological process of osteoarthritis (OA). Herein, we investigated the protective effect of moderate mechanical stress on chondrocyte ferroptosis and further revealed the internal molecular mechanism. Intra-articular injection of sodium iodoacetate (MIA) was conducted to induce the rat model of OA in vivo, meanwhile, interleukin-1 beta (IL-1β) was treated to chondrocytes to induce the OA cell model in vitro. The OA phenotype was analyzed by histology and microcomputed tomography, the ferroptosis was analyzed by transmission electron microscope and immunofluorescence. The expression of ferroptosis and cartilage metabolism-related factors was analyzed by immunohistochemical and Western blot. Animal experiments revealed that moderate-intensity treadmill exercise could effectively reduce chondrocyte ferroptosis and cartilage matrix degradation in MIA-induced OA rats. Cell experiments showed that 4-h cyclic tensile strain intervention could activate Nrf2 and inhibit the NF-κB signaling pathway, increase the expression of Col2a1, GPX4, and SLC7A11, decrease the expression of MMP13 and P53, thereby restraining IL-1β-induced chondrocyte ferroptosis and degeneration. Inhibition of NF-κB signaling pathway relieved the chondrocyte ferroptosis and degeneration. Meanwhile, overexpression of NF-κB by recombinant lentivirus reversed the positive effect of CTS on chondrocytes. Moderate mechanical stress could activate the Nrf2 antioxidant system, inhibit the NF-κB p65 signaling pathway, and inhibit chondrocyte ferroptosis and cartilage matrix degradation by regulating P53, SLC7A11, and GPX4.

## Introduction

Osteoarthritis (OA) is the most common chronic degenerative bone joint disease characterized by cartilage degeneration, destruction, and bone hyperplasia. The main clinical manifestations are joint swelling, pain, and limited movement, which have become the main cause of disability in elderly people^1^. Recently, the incidence of OA has increased rapidly because of aging and obesity, which affecting approximately 302 million people worldwide and causing a massive disease burden^2^. However, there is still no effective treatment. At present, the clinical treatment of OA primarily includes surgery, drugs, and physical therapy. Surgery is costly and invasive, and drugs have evident side effects^3^. Hence, physical therapy is increasingly recommended by the guideline for the management of OA^4^.

Exercise is strongly recommended for patients with OA. Accumulating evidence has demonstrated that regular moderate aerobic exercise in patients with OA can effectively relieve joint swelling, pain, function, and improve quality of life^5,6^. Moderate mechanical stress is essential to maintain cartilage homeostasis and normal function, it can protect articular cartilage, inhibit chondrocyte inflammatory response and programmed cell death, delay the progression of OA^7,8^. Chondrocytes are mechanosensitive cells that can convert mechanical signals into biochemical signals and participate in regulating various biological processes of chondrocytes. They are closely related to cartilage homeostasis and repair^9^. Moreover, mechanical stress could inhibit chondrocyte autophagy and apoptosis^10,11^, however, whether or not mechanical stress is related to chondrocyte ferroptosis has not been studied.

Ferroptosis is a newly discovered type of programmed cell death that is primarily characterized by excess iron accumulation and lipid peroxidation^12^. Iron metabolism and iron homeostasis play vital roles in the human pathophysiological process, and abnormal iron metabolism is closely associated with the pathophysiological processes of musculoskeletal diseases, such as osteoporosis^13^, arthropathies^14^, and osteosarcoma^15^. Recently, ferroptosis has become a hot topic in the study of bone and joint diseases. Studies found that ferroptosis occurs in chondrocytes in OA, plays an important role in the pathophysiological process of OA^16–19^. Nuclear factor E2-related factor 2 (Nrf2), glutathione peroxidase 4 (GPX4), solute carrier family 7 member 11 (SLC7A11) and P53 are key regulator of ferroptosis. GPX4, SLC7A11, Nrf2 act as ferroptosis inhibitors and P53 as a ferroptosis promoter, participate in the biological process of ferroptosis by regulating antioxidant system and lipid ROS formation^12,20^. However, the mechanisms of activation and regulation of ferroptosis in OA remains unclear. The activation of the nuclear factor-κB (NF-κB) signaling pathway could regulate ferroptosis and participate in the occurrence and development of tumors and nervous system diseases^21,22^. However, whether or not NF-κB signaling pathways regulate ferroptosis in OA, and whether or not the molecular mechanism of mechanical stress alleviates cartilage degeneration is related to ferroptosis needs to be further explored.

Therefore, in this study, treadmill exercise was carried out on OA rats, and cyclic tensile strain (CTS) was taken for osteoarthritis chondrocytes. We will explore the influence of mechanical stress on chondrocyte ferroptosis (mainly include GPX4, SLC7A11, P53, Nrf2) and cartilage degeneration. Then, the potential regulatory mechanism of ferroptosis was investigated, mechanical stress inhibited NF-κB p65 signaling pathways thereby restraining chondrocyte ferroptosis and degradation. In conclusion, this study provides new ideas and directions for the molecular mechanism study of exercise therapy for OA.

## Materials and methods

### Osteoarthritis rat model and treadmill protocols

The experimental Sprague–Dawley rats were purchased from SPF Biotechnology Co., Ltd. (Beijing, China), and fed under constant temperature and humidity conditions. Temperature and humidity were 22 ± 3 °C and 40–60%, respectively. The room was illuminated by alternating day and night in 12/12 h. The study was approved by the Ethics Committee of Shenyang Sport University.

A total of 40 male SD rats (200 ± 20 g) were randomly divided into four groups (n = 10): control group (CON), exercise control group (CONE), osteoarthritis model group (OA), and modeling exercise group (OAE). Before the intervention, all the rats underwent adaptive treadmill exercise for 5 days, and the exercise intensity gradually increased from 0 to 10 m/min, 10 min/day^23^. The treatments were as follows: (1) The rats from the CON group were injected with 50 μl physiological saline inside the right knee joint and fed conventionally without special intervention. (2) Meanwhile, the CONE group had an intra-articular injection of 50 μl physiological saline, and the treadmill exercise was performed 24 h later. Based on previous experiments and related studies, the moderate-intensity treadmill exercise intensity of rats was 18 m/min, 30 min/day, and 5 d/week for 4 weeks^24^. (3) Rats from the OA group were injected in the right intra-articular joint with 1 mg/50 μl of sodium iodoacetate (MIA) to induce the rat model of OA^8,25^, the rats were fed conventionally without special intervention. (4) The KOA group was injected with 1 mg/50 μl of MIA (57858, Sigma, USA) to induce an OA model, and after 24 h of knee joint injection, a 4-week treadmill exercise intervention was performed. During the intervention, the joint diameter was measured weekly for all rats. All animal experiments were approved by Ethics Committee of Shenyang Sport University (protocol code 20212). All the procedures followed the rules of the International Council for Laboratory Animal Science for the protection of the laboratory animals employed for scientific purposes and complied with the ARRIVE guidelines (Animal Research: Reporting In Vivo Experiments). Utmost efforts were made to decrease animal suffering as well as the number of animals utilized in current study.

### Microcomputed tomography

At the end of 4 weeks treadmill exercise intervention, all rats were euthanized under deep isoflurane anesthesia. Microcomputed tomography (micro-CT) was used to observe the surface of tibia as well as trabecular and cortical areas. Briefly, after fixation in 4% paraformaldehyde 48 h, the right knee joint was scanned in Scanco Viva CT40 (Scanco Medical, Bassersdorf, Switzerland) for microstructure analysis^26^. The main scanning parameters are 65 kV, 385 mA, 10 mm voxel size, and 300 ms exposure. The measured parameters were as follows: (1) SMI; (2) BMD, mg/cm3; (3) porosity (total porosity, %); (4) BV/TV, %; (5) Conn.Dn, 1/mm^3^; (6) Tb.Pf, 1/mm; (7) Tb.SP, mm; (8) Tb.Th, mm; and (9) Tb.N, 1/mm. Scanco analysis software was used to analyze all the data^11,27^.

### H&E staining

H&E staining was used to observe the histologic parameters of articular cartilage and subchondral bone, and further used Mankin’s score to assess the histopathologic grade of cartilage. Briefly, right knee joints were fixed for approximately 48 h with 4% paraformaldehyde, after 4 h of running water, the fixed knee joints were placed into the decalcifying solution (ZLI-9307, ZSGB-BIO, China) for 3 days to decalcify. Then embedded, sliced, dewaxed, and rewatered, hematoxylin and eosin dyes were dripped for 5 min and 3 min, respectively. After washing with tap water, gradient dehydration and transparency were performed, and finally the neutral gum was used for sealing.

### Toluidine blue staining

The experiment was conducted based on the instructions of the Toluidine Blue Cartilage Chain Kit (G2543, Solarbio, China). The slices were soaked in cartilage stain solution for approximately 30 min. Then washed in running water for 2 min. Subsequently, ethanol was dehydrated and transparent and then sealed with neutral gum. OARSI score was used to assess the degree of cartilage damage.

The cell morphology of cartilage and subchondral bone was independently observed and scored by two observers using Mankin’s and OARSI scoring systems. The average value of the two observers was taken as a statistical value to systematically evaluate the degree of cartilage degeneration in the OA model.

### Immunohistochemical

After the sections were deparaffinized and rehydrated, the experiments were performed following the IHC kit instructions (kit-9720, MXB, China) for MMP13 (18165-1-AP; 1:200; Proteintech), Nrf2 (bs-1074R; 1:500; Bioss), P53 (A0263; 1:100; Abclonal), SLC7A11 (DF12509; 1:100; Affinity Biosciences), Col2a1 (28459-1-AP; 1:1000; Proteintech), p-NF-κB p65 (bs-5662R; 1:200; Bioss), and GPX4 (67763-1-Ig; 1:2000; Proteintech). A series of primary antibodies were added to the sections of the four groups, and then all sections were incubated in a wet box at 4 °C overnight. The following day, the sections were incubated at room temperature for 1 h with a secondary antibody, and a DAB solution (dab-0031, MXB, China) was added for visualization. Finally, after neutral resin blocking, they were photographed under a microscope. Five fields were randomly selected for each sample, and the expression of each index was examined and averaged for statistical analysis.

### Osteoarthritis cell model

Human normal chondrocyte C28/I2 (Cell Bank of the Chinese Academy of Sciences, China) was cultured in DMEM/F12 (Geneze, China) with 10% fetal bovine serum (Gibco, USA) and 1% penicillin–streptomycin (Gibco, USA), and maintained in a wet incubator filled with 5% CO_2_ at 37 °C. OA cell model was introduced by interleukin-1 beta (IL-1β)^28,29^. Briefly, C28/I2 cells were inoculated into 6-well plates at a density of 9 × 10^4^ cells/well, after 24 h of cell culture, the cells were starved for 12 h, then 10 ng/ml IL-1β (211-11B, Peprotech, USA) was added for 24 h.

### Mechanical stress stimulation

Mechanical stress was applied to chondrocytes by FX-2000 Flexcell system^®^ (Flexcell International, McKeesport, PA, USA). The FX-2000 Flexcell system could create a cyclic tensile strain for cultured cells to simulate mechanical stress stimulation during body movement, which is widely used in basic research^30,31^. Chondrocytes were inoculated into 6-well BioFlex^®^ culture plates which were precoated with plate collagen I at a density of 9 × 10^4^ cells/well. CTS was enforced at 5% intensity, 0.5 HZ, and 1/2 sine^32^. Based on different experimental requirements, the cells were exposed to CTS for 2 h, 4 h, and 8 h with or without IL-1β induction.

### Western blot analysis

BCA kit (P0012, Beyotime, China) was used to quantify the extracted protein. After quantification, it was then electrophoresed in 10% SDS-PAGE gel and transferred to a PVDF membrane (Millipore, USA). After blocking with 5% skim milk, the primary antibody was incubated at 4 °C overnight, and the secondary antibody was incubated at room temperature for 1 h. ECL kit (Tanon, China, 185001) and chemiluminescence instrument (Tanon, China) were used for imaging. The primary antibodies against were listed as follows: MMP13 (18165-1-AP; 1:2000; Proteintech), MMP3 (A11418; 1:1000; Abclonal), Col2a1 (ab307674; 1:1000; Abcam), ADAMTS5 (bs-3573R; 1:1000; Bioss), GPX4 (67763-1-Ig; 1:1000; Proteintech), Nrf2 (ab307026; 1:5000; Abcam), P53 (ab26; 1:500; Abcam), SLC7A11 (ab175186; 1:5000; Abcam), NF-κB p65 (8242T; 1:1000; CST), p-NF-κB p65 (3035; 1:1000; CST), and GAPDH (A19056; 1:10000; Abclonal).

### Immunofluorescence

Chondrocytes were inoculated into 12-well at an intensity of 4 × 10^4^ cells/well, after IL-1β and CTS intervention, the cells were added Mito-Tracker Deep Red 633 (C1034, Beyotime, China) for 30 min for mitochondrial staining, then fixed with 4% paraformaldehyde for 20 min, 0.5% TritonX-100 (T8200, Solarbio, China) for 20 min, 5% donkey serum (S9100, Solarbio, China) for 1 h, the primary antibody GPX4 (67763-1-Ig; 1:500; Proteintech) or NF-κB p65 (8242T; 1:500; CST) was incubated at 37 °C for 4 h, and Alexa Fluor 555 (A0453, Beyotime, China,) was incubated at 37 °C for 2 h. DAPI (C1005, Beyotime, China) was nuclear stained for 10 min and then photographed under the fluorescence microscope (Nikon, Japan).

### Transmission electron microscopy (TEM)

The TEM was used to assess the mitochondrial morphological changes of ferroptosis. Cells were washed twice and centrifuged for 5 min at 1000 rpm to harvest all the cells. Then, the cold 2.5% glutaraldehyde was used to fix the cells at 4 °C for approximately 12 h. Before dehydration, cells were fixed again with 1% osmium acid and then embedded in epoxy resin. The ultrathin sections were further stained with lead citrate and uranyl acetate and finally examined by TEM (JEM-1400Plus, Tokyo, Japan).

### Cell viability assays

Human chondrocytes were seeded onto 96-well plates overnight with 8 × 10^3^ cells/well. The following day, different concentrations (2.5, 5, 10, 20 μM) of NF-κB inhibitor BAY 11-7082 (Catalog No. S2913, Selleck, USA) were added and continued to incubate for 12 and 24 h. Cell Counting Kit-8 (CCK-8) is a simple and sensitive cell viability assay. A 10 µl CCK-8 (Dojindo, Kumamoto, Japan) solution was added to each well at 37 °C for 2 h, then absorbance values were measured at 450 nm to calculate the cell survival rate. The experiment was repeated thrice.

### Quantitative reverse-transcription PCR (qRT-PCR)

Human chondrocytes were seeded onto 6-well plates with 7 × 10^4^ cells/well for 24 h. Then, different concentrations (10, 20, 40 MOI) NF-κB p65 overexpresses recombinant lentivirus (Shanghai Genechem Co., Ltd) were added and continued to incubate for 16 h. Then, the medium containing lentivirus was discarded, replaced with a new complete medium, and continued incubation for 3 days. Total RNA was extracted by Total RNA Extraction Reagent (Catalog No. R401, Vazyme, China). The RNA concentration was measured via NanoDrop 2000 (Thermo, Waltham, USA), and cDNA was prepared by ABScript III RT Master Mix for qPCR with gDNA Remover (Catalog No. RK20429, Abclonal, China). qRT-PCR was performed by 2X Universal SYBR Green Fast qPCR Mix (Catalog No. RK21203, Abclonal, China) in Real-Time System (Thermo, Waltham, USA). Relative gene expression was normalized by GAPDH using the 2^−ΔΔCt^ method. The primers were ordered from Sangon Biotech. GAPDH (forward primer 5′-AGA AGG CTG GGG CTC ATT TG-3′, reverse primer 5′-AGG GGC CAT CCA CAG TCT TC-3′). NF-κB p65 (forward primer 5′-TAT CTC GCT TTC GGA GGT GC-3′, reverse primer 5′-GCG TGG AGG AAG ACA CTT GA-3′).

### Statistical analysis

All data were expressed as mean ± standard error (mean ± SD) and analyzed by SPSS 23.0. Before the statistical analysis of all data, Shapiro–Wilk and Levene’s test was used to analyze whether or not the data conformed to normal distribution or homogeneity of variance. One-way ANOVA was used for data consistent with normal distribution and homogeneity of variance and LSD for the post hoc test; otherwise, the rank sum test was used. GraphPad Prism 8.0.2 software was used to draw statistical pictures, such as histograms and line graphs. *P* < 0.05 was considered statistically significant.

### Ethics approval and consent to participate

All animal experiments were approved by Ethics Committee of SHENYANG SPORT UNIVERSITY (protocol code 20212).

## Results

### Treadmill exercise alleviated the progression of osteoarthritis in rats

**Figure 1:**
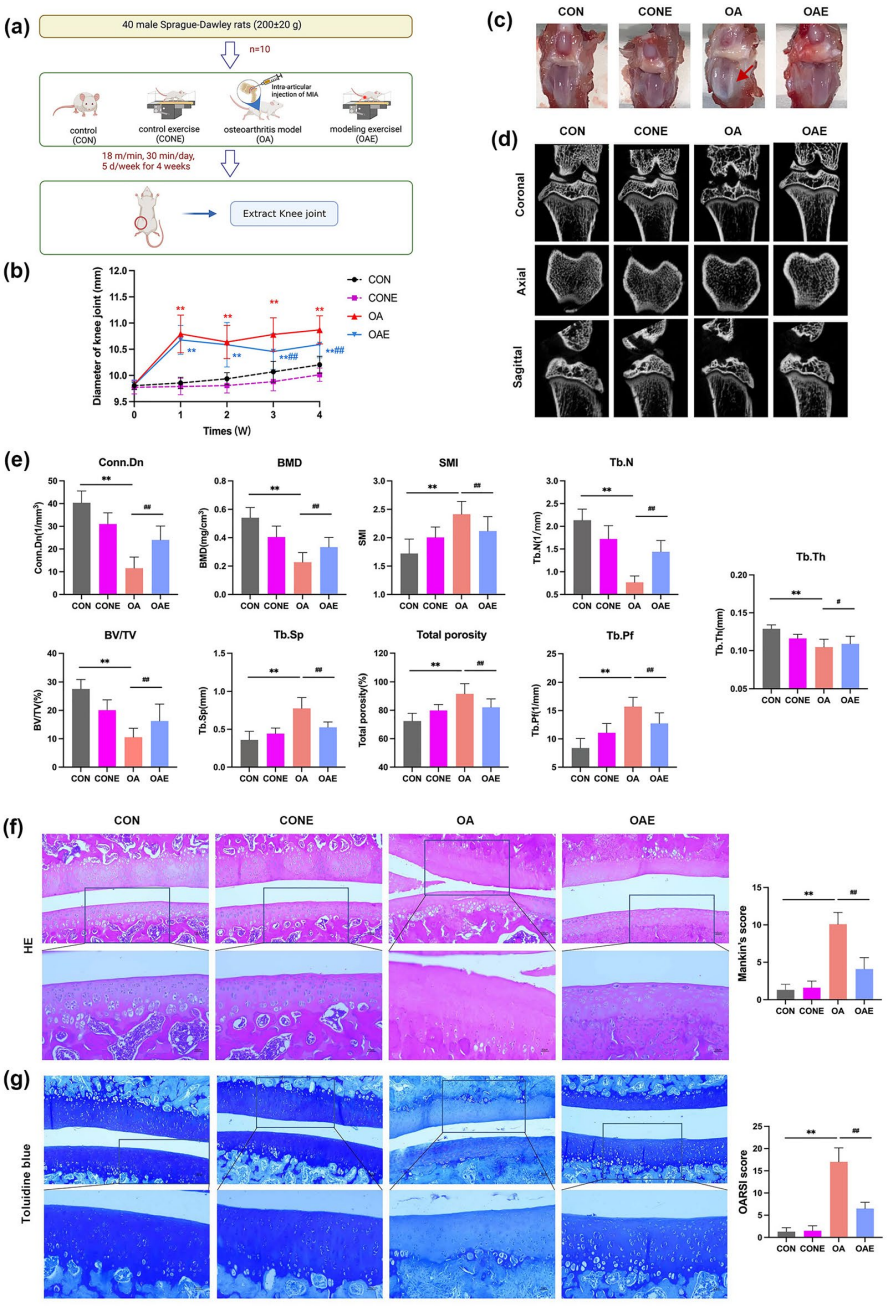
Treadmill exercise inhibited cartilage degradation in rats with knee osteoarthritis. (**a**) Animal experiment design, n = 10 rats per group. (**b**) Diameter of the knee joint in each group. (**c**) Gross imaging of the different groups. (**d**) The microcomputed tomography of the knee joint in each group. The scanning angles were coronal, sagittal, and transverse planes. (**e**) The microstructure analysis of microcomputed tomography, the figure shows the statistics of measured parameters bone mineral density (BMD), bone volume fraction (BV/TV), connection density (Conn.Dn), number of bone trabeculae (Tb.N), trabecular space (Tb.SP), trabecular thickness (Tb.Th), trabecular pattern factor (Tb.Pf), structural model index (SMI), and porosity. (**f**) H&E staining and Mankin’s score of the knee joint in each group. (**g**) Toluidine blue staining and OARSI score of the knee joint in each group. The upper was ×100 with scale bar 100 μm and lower was ×200 with scale bar 50 μm in (**f**,**g**). **P < 0.01 compared with CON; ^#^*P* < 0.05, ^##^*P* < 0.01 compared with OA, n = 5 per group.

The treadmill exercise intervention was performed on MIA-induced rats to explore the effects of exercise on knee osteoarthritis. The grouping and treatment were shown in Fig. 1a. Joint pain and swelling are the basic symptoms of early OA. The keen joints of OA were significantly swollen after being injected with MIA as compared with the CON group (*P* < 0.01), but gradually subsided by treadmill exercise intervention. In the 3rd week, the swelling of rats in the OAE group was significantly lower than OA group (*P* < 0.01; Fig. 1b). The joint surface of normal rats (CON and CONE groups) was complete and smooth with a small amount of hyaline joint fluid, and the cartilage surface of OA (OA group) was swollen and degenerated, and the color was changed. After the treadmill exercise, the cartilage damage was significantly relieved (OAE group; Fig. 1c). The result of micro-CT showed that there was a significant osteophyte formation in the OA group, accompanied decreased bone density and bone mass. However, bone damage was significantly improved after treadmill exercise (Fig. 1d). Significant differences were observed in bone mineral density (BMD), bone volume fraction (BV/TV), connection density (Conn.Dn), number of bone trabeculae (Tb.N), trabecular space (Tb.SP), trabecular thickness (Tb.Th), trabecular pattern factor (Tb.Pf), structural model index (SMI), and porosity as compared with CON or OA (*P* < 0.01 or *P* < 0.05; Fig. 1e). H&E (Fig. 1f) and toluidine blue staining (Fig. 1g) showed that the cartilage surface of the normal rat was smooth without cracks and regular cell arrangement, whereas the OA cartilage surface was rough and irregular, and the cartilage thickness was reduced. After the treadmill exercise, the cartilage damage was significantly improved, and the Mankin’s and OARSI scores showed a similar trend. Those results combined indicated that treadmill exercise can effectively relieve the progression of OA in rats.

### Treadmill exercise alleviated cartilage matrix degradation of osteoarthritis

**Figure 2:**
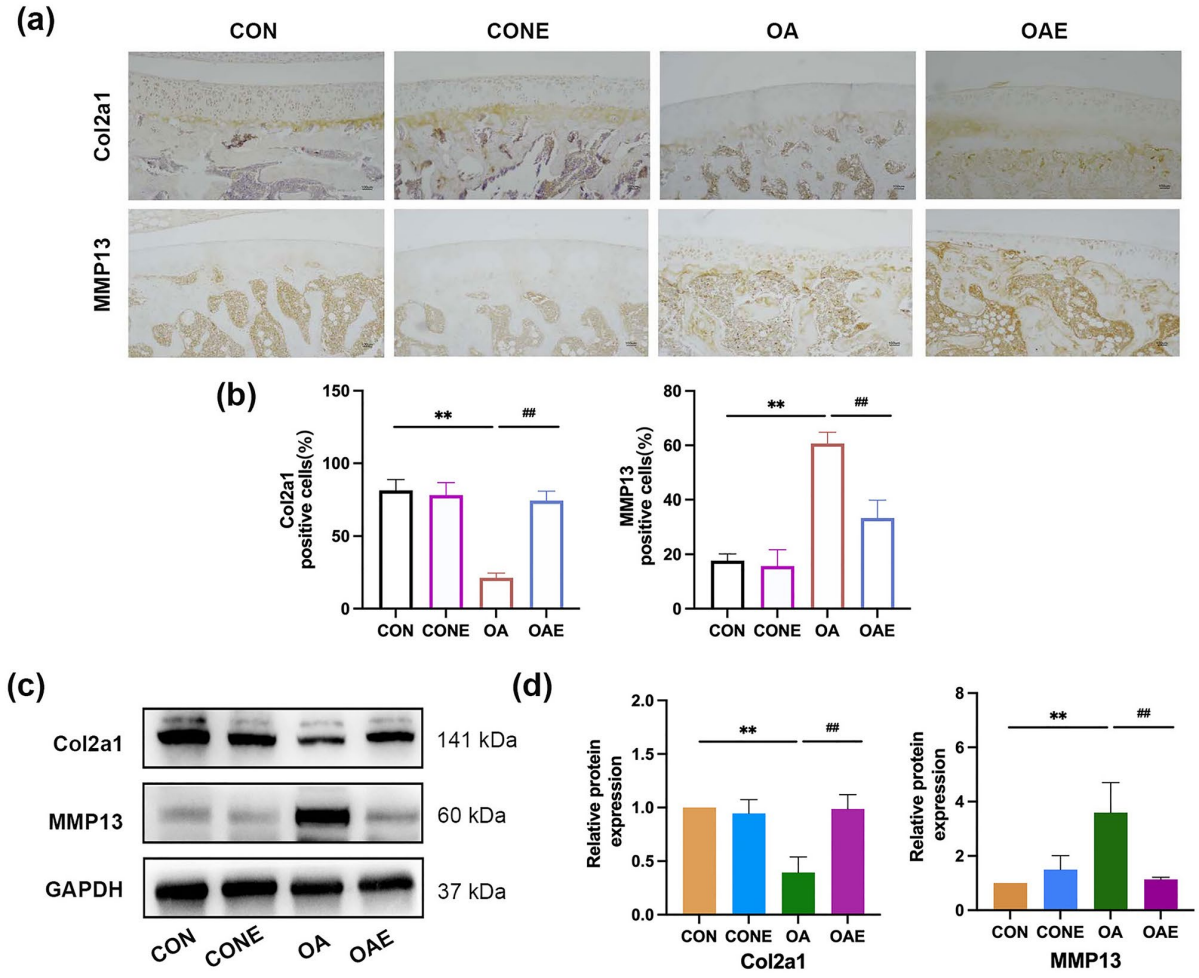
Treadmill exercise inhibited cartilage matrix degradation in rats with knee osteoarthritis. (**a**) Immunohistochemical of Col2a1 and MMP13 in knee joint, and the staining results were calculated by the percentage of positive staining cells (**b**), ×100, Scale bar, 100 μm. (**c**) Results of Western blot analysis of articular cartilage in each group, and the relative protein expression level was calculated in (**d**). **P < 0.01 compared with CON; ^##^*P* < 0.01, compared with OA, n = 5 per group.

MMP13 and Col2a1 are commonly used indicators to evaluate the degradation of the cartilage matrix^33,34^. The result of immunohistochemical showed that the number of positively stained cells of Col2a1 in the OA group was significantly decreased, the number of MMP13 was significantly increased. There was a significant difference as compared with the CON group (*P* < 0.01). Furthermore, the expression of Col2a1 was significantly upregulated and MMP13 was downregulated after treadmill exercise (*P* < 0.01; Fig. 2a,b). The results of Western blot were similar to those of immunohistochemistry (Fig. 2c,d). The studies above confirmed that treadmill exercise can effectively relieve cartilage matrix degradation of osteoarthritis in rats.

### Treadmill exercise inhibited ferroptosis in rats with knee osteoarthritis

**Figure 3:**
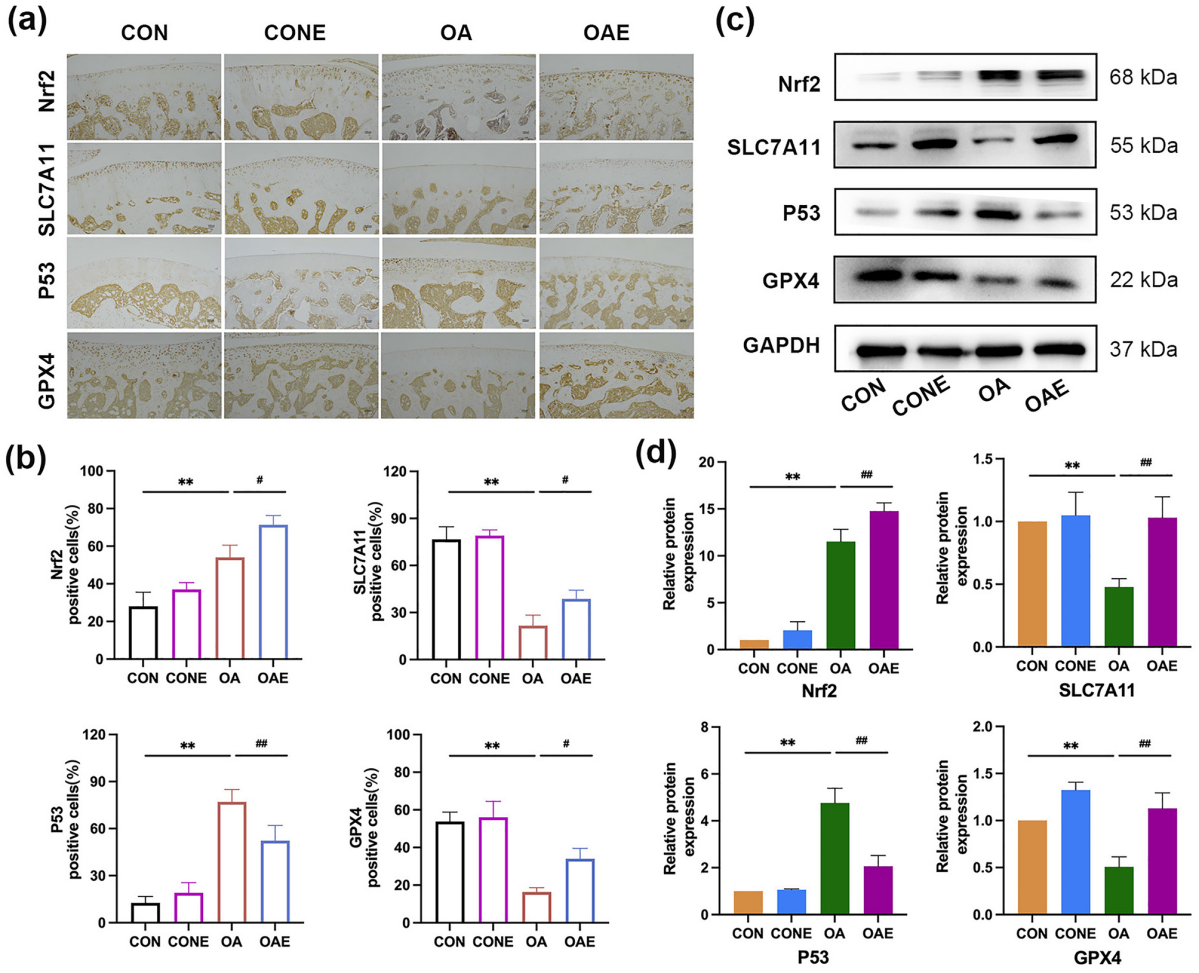
Treadmill exercise inhibited ferroptosis in rats with knee osteoarthritis. (**a**) Immunohistochemical of Nrf2, P53, SLC7A11, and GPX4 in knee joint, the staining results were calculated by the percentage of positive staining cells (**b**), ×100, Scale bar, 100 μm. (**c**) Results of Western blot analysis of articular cartilage in each group, and the relative protein expression level was calculated in (d). **P < 0.01 compared with CON; ^##^*P* < 0.01, ^#^*P* < 0.05 compared with OA, n = 5 per group.

Ferroptosis is crucial in the pathophysiological processes of various diseases^12,35^. In this study, we initially conducted animal experiments to confirm the role of ferroptosis in the pathogenesis of OA and explorer whether or not the mechanism of exercise alleviating OA is related to ferroptosis. The result of immunohistochemical showed that numerous brown stained cells of SLC7A11 and GPX4 were observed in normal cartilage, while they were decreased in OA (*P*＜0.01), and the number of positive staining cells increased after treadmill exercise (*P*＜0.05). On the contrary, P53 was upregulated in OA, and the expression was decreased after treadmill exercise (*P* < 0.01). Nrf2 was downregulated in normal cartilage and upregulated in OA, however, the expression of Nrf2 was further increased after exercise intervention (*P* < 0.05 or *P* < 0.01; Fig. 3a,b). Consistently, the results of Western blot were similar to those of immunohistochemistry (Fig. 3c,d). The studies above demonstrated that ferroptosis contributes to the progression of OA, and treadmill exercise can activate Nrf2, and inhibit the progression of ferroptosis by regulating P53, SLC7A11, and GPX4.

### Mechanical stress inhibited IL-1β-induced chondrocyte degradation

**Figure 4:**
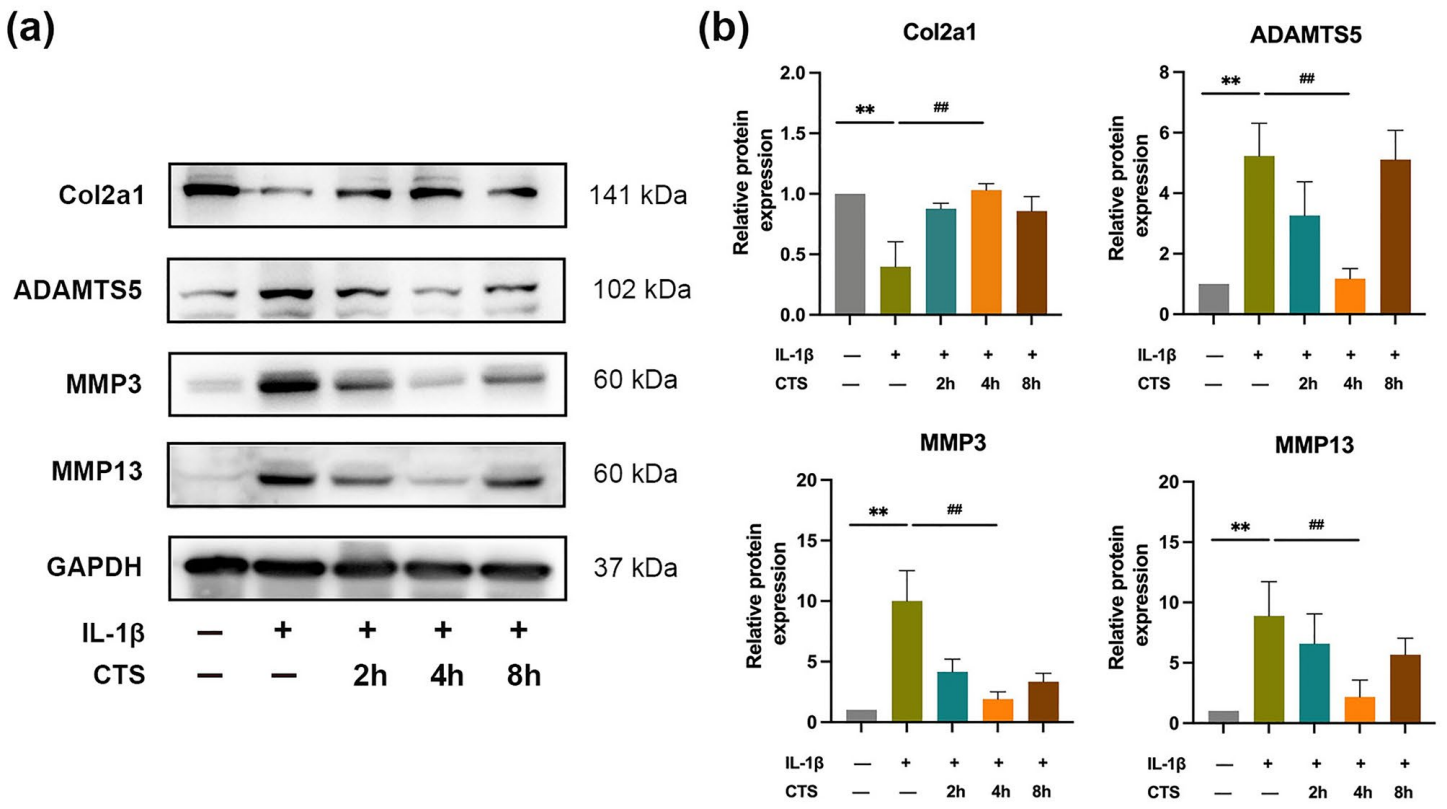
Mechanical stress protected IL-1β-induced chondrocyte degradation. (**a**) Effect of cyclic tensile strain (CTS) at different times on chondrocytes in osteoarthritis, the relative protein expression level was calculated in (**b**).**P < 0.01 compared with control; ^##^*P* < 0.01 compared with IL-1β group, the experiment was repeated thrice.

Herein, cell experiments were conducted in vitro to explore the underlying mechanism that exercise inhibits ferroptosis and improves osteoarthritis. First, the positive effect of mechanical stress on chondrocytes in OA was determined. We used human normal chondrocyte C28/I2, the OA cell model was introduced by IL-1β (10 ng/ml for 24 h), and mechanical stress was applied to chondrocytes by CTS (5% intensity, 0.5 HZ, 1/2 sine). The expression of cartilage catabolic-related factors (MMP3, MMP13, and ADAMTS5) was increased and Col2a1was decreased after IL-1β stimulation (*P* < 0.01), however, the expression significantly changed by 4 h CTS intervention (*P* < 0.01; Fig. 4a,b). Therefore, 4 h CTS is the appropriate stimulus intensity and can be used in follow-up experiments. In summary, studies above indicated that 4 h CTS can inhibit OA chondrocyte degradation by regulating Col2a1 and MMP13.

### Mechanical stress inhibited IL-1β-induced chondrocyte ferroptosis

**Figure 5:**
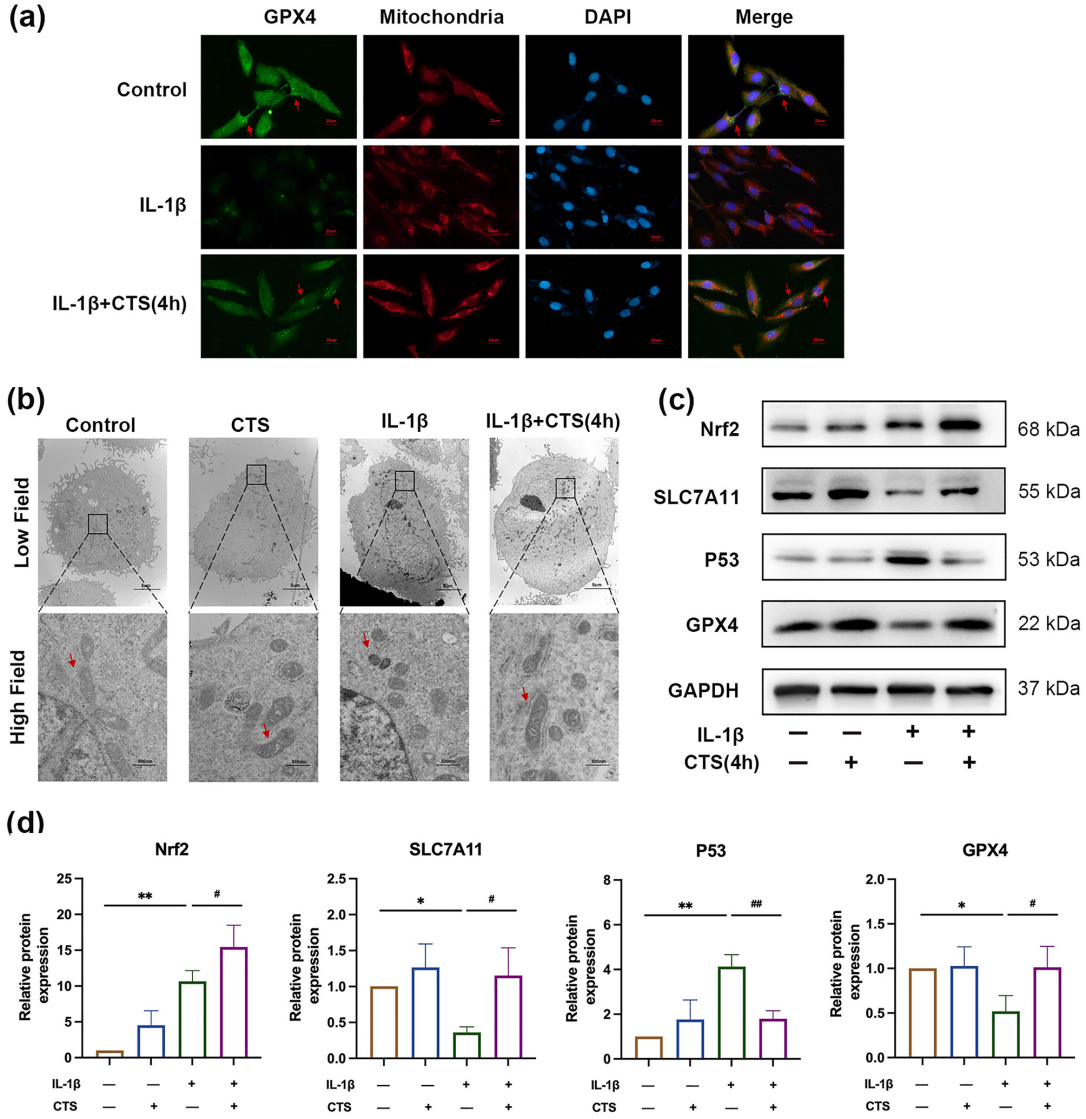
Mechanical stress relieved IL-1β-induced chondrocyte ferroptosis. (**a**) Immunofluorescence staining of GPX4 and mitochondrial in chondrocyte with IL-1β and/or CTS. GPX4 staining is green, mitochondrial staining is red, and the red arrows are GPX4 positive staining. ×400, Scale bar, 25 μm. (**b**) Transmission electron microscopy of chondrocytes with IL-1β and/or CTS. The red arrows are normal mitochondria or mitochondria with abnormal morphology induced by ferroptosis, which primarily manifests as shrinkage of mitochondria with increased membrane density and reduction in or vanishing of mitochondrial cristae. Low field ×6000, scale bar, 5 μm; high field ×40,000, scale bar, 500 nm. (**c**) Effect of 4 h CTS on IL-1β-induced ferroptosis-related factors in chondrocyte, the relative protein expression level was calculated in (**d**). *P < 0.05, **P < 0.01 compared with control; ^##^*P* < 0.01, ^#^*P* < 0.05 compared with IL-1β group, the experiment was repeated three times.

Immunofluorescence, transmission electron microscopy, and ferroptosis-related factors (Nrf2, P53, SLC7A11, and GPX4) were used to determine whether or not ferroptosis occurred in OA chondrocytes and whether or not ferroptosis was related to the mechanism of mechanical stress. GPX4 is the key regulator of the occurrence of ferroptosis, mitochondrial isoform of GPX4 is thought to be localized in mitochondria^36^. Thus, inhibiting GPX4 can accelerate ferroptosis^37^. Results of immunofluorescence showed that mitochondrial GPX4 was upregulated in normal chondrocytes, and it was downregulated in IL-1β-induced chondrocytes, 4 h CTS reversed the expression of GPX4 (Fig. 5a). The most notable morphological features of ferroptosis cells are mitochondrial atrophy and increased membrane density, as well as the reduction of mitochondrial cristae^38^. As shown in Fig. 5b, mitochondria atrophy with elevated membrane density were observed in IL-1β-induced chondrocytes, and mitochondrial morphology was recovered after CTS. The result of Western blot analysis revealed that IL-1β activated Nrf2, decreased the expression of ferroptosis-inhibiting factors GPX4 and SCL7A11, and increased the expression of ferroptosis-promoting factor P53 (*P* < 0.01). Then, 4 h CTS intervention further activated Nrf2 and reversed the expression of SCL7A11, GPX4, and P53 (*P* < 0.01; Fig. 5c,d). In short, the results above are similar to those of animal experiments, ferroptosis contributes to the degeneration of OA chondrocytes, and CTS can activate Nrf2 and inhibit the progression of ferroptosis by regulating the expression of the key ferroptosis-regulators.

### Exercise alleviated the progression of osteoarthritis by inhibiting the NF-κB p65 signaling pathway

**Figure 6:**
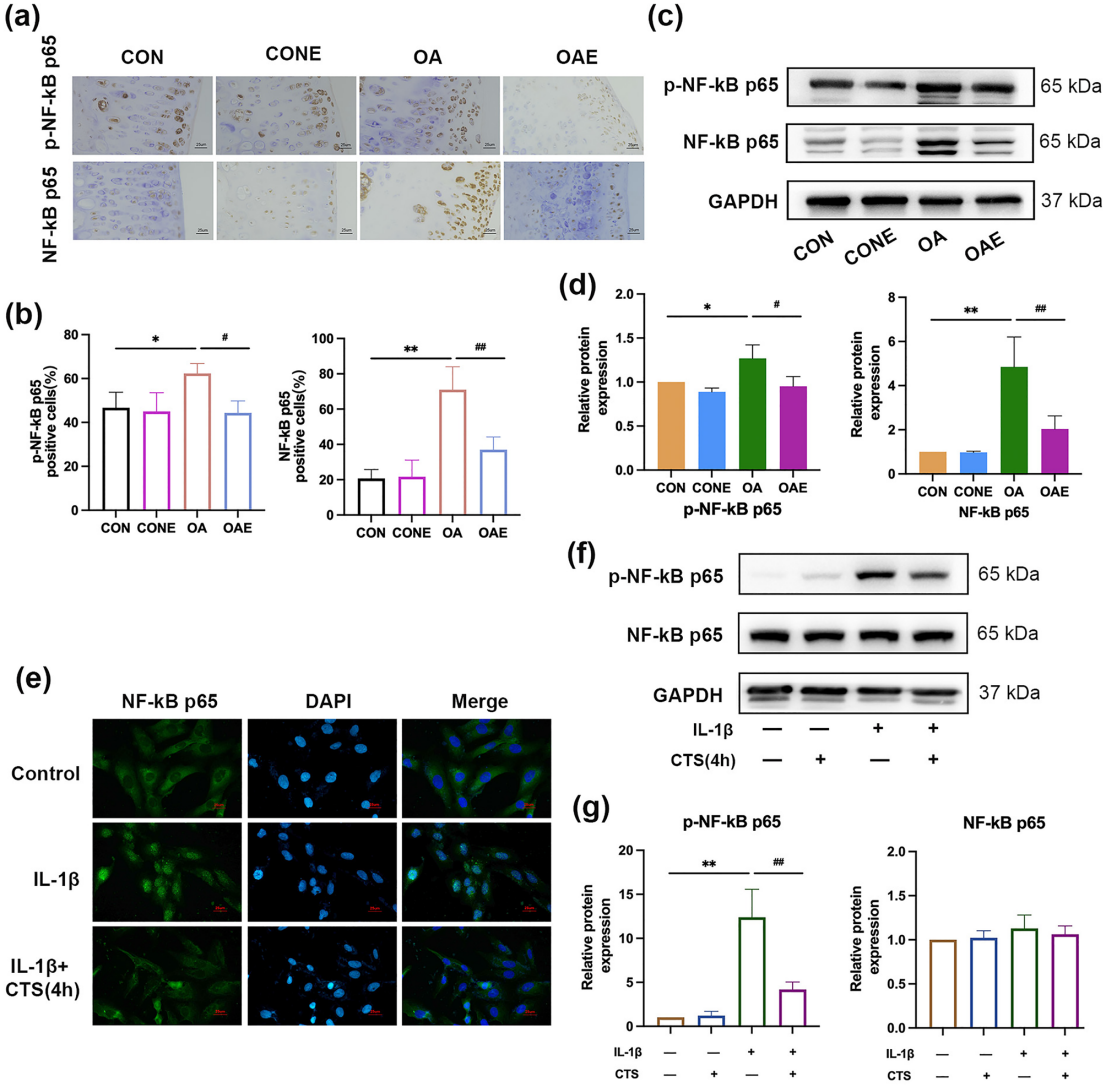
Expression of the NF-κB p65 signaling pathway in osteoarthritis with/without exercise intervention. (**a**) Expression of NF-κB p65 in the cartilage of osteoarthritis rats by immunohistochemical. Treadmill exercise intervention was performed on the MIA-induced osteoarthritis rat model and immunohistochemical analysis of the percentage of NF-κB p65 positive cells (**b**). ×400, scale bar, 25 μm. n = 5. (**c**) Protein expression of NF-κB p65 in the cartilage of osteoarthritis rats by Western blot analysis. The relative protein expression level was calculated in (**d**). n = 5. (**e**) Expression of NF-κB p65 in chondrocyte treated with IL-1β and/or CTS by immunofluorescence staining. Osteoarthritis cell model was introduced by IL-1β in human chondrocyte C28/I2, and mechanical stress was applied by CTS. The NF-κB p65 expression was detected by immunofluorescence staining. ×400, scale bar, 25 μm. (**f**) Protein expression of NF-κB p65 in chondrocytes treated with IL-1β and/or CTS by Western blot. The relative protein expression level was calculated in (**g**), and the experiment was repeated thrice. **P < 0.01 compared with CON (or control); ^##^*P* < 0.01, compared with OA (or IL-1β group).

NF-κB p65 is a key regulator of ferroptosis in cancer^39,40^. It also acts as a promoter accelerating the occurrence and development of osteoarthritis^41^. Therefore, we hypothesized that the NF-κB signaling pathway may play an essential role in the mechanism of exercise that alleviates ferroptosis in OA. Thus, we first examined the expression of NF-κB p65 in animals and cell models of OA treated with treadmill exercise (or mechanical stress) to verify the hypothesis. Results of immunohistochemical (Fig. 6a,b) and Western blot assays (Fig. 6c,d) in the cartilage of OA rats showed that NF-κB p65 signaling pathway was activated in OA rats (*P* < 0.01), treadmill exercise intervention inhibited the expression of NF-κB p65 (*P* < 0.01). Similar results were observed in cell experiments. Results of immunofluorescence staining showed that NF-κB p65 was downregulated and primarily located in the cytoplasm in normal chondrocytes, however, the expression is significantly increased in the nucleus by IL-1β induction. 4 h CTS inhibited the activation of NF-κB p65 (Fig. 6e). In addition, NF-κB p65 signaling pathway was activated in IL-1β-induced chondrocytes, 4 h CTS reversed the phosphorylation of NF-κB p65 (Fig. 6f,g). In summary, studies above indicated that exercise alleviated the progression of osteoarthritis by inhibiting the NF-κB p65 signaling pathway.

### Mechanical stress relieved chondrocyte degradation and ferroptosis by inhibiting the NF-κB p65 signaling pathway

**Figure 7:**
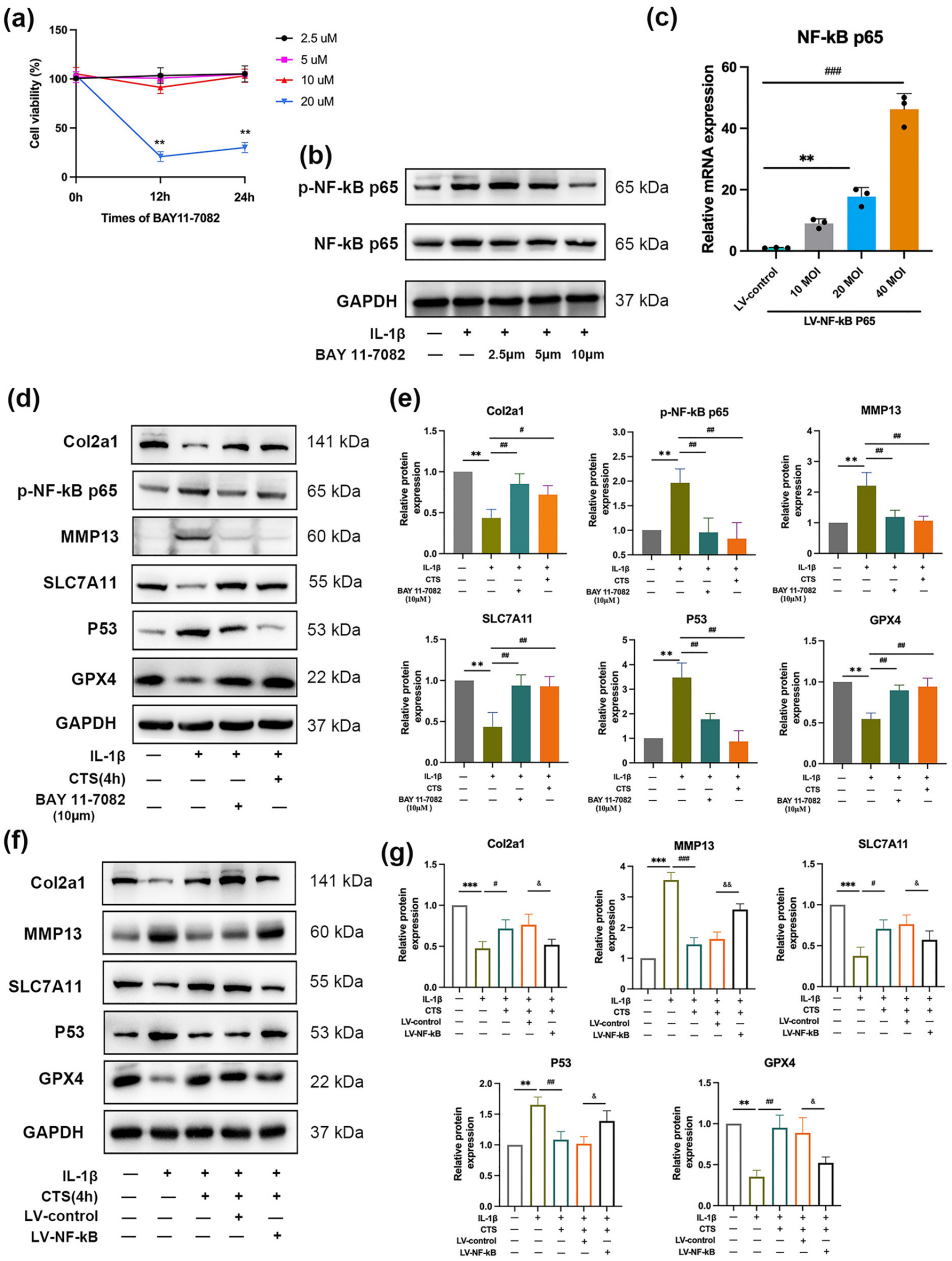
Effect of NF-κB pathway inhibition and overexpression on ferroptosis and degradation of osteoarthritis chondrocyte. (**a**) Effect of BAY 11-7082 on chondrocyte’s cell activity at different concentrations and times. C28/I2 cells were incubated with different concentrations (2.5, 5, 10, and 20 μM) of NF-κB inhibitor (BAY 11-7082) for 12 h and 24 h respectively, and CCK8 assay was used to detect cell activity. (**b**) Effect of different concentrations of BAY 11-7082 on NF-κB expression in chondrocytes. C28/I2 cells were incubated with different concentrations of BAY 11-7082 (2.5, 5, 10 μM) with/without IL-1β for 24 h, the expression of NF-κB p65 was detected by Western blot. (**c**) The expression of NF-κB p65 in chondrocytes with different concentrations of NF-κB overexpression recombinant lentivirus. Chondrocytes at logarithmic growth stage were incubated with recombinant lentivirus at different concentrations (10, 20, 40 MOI) for 72 h, then qRT-PCR was used to detect the mRNA expression of NF-KB p65. (**d**) Effect of BAY 11-7082 on ferroptosis and degradation of osteoarthritis chondrocyte. C28/I2 cells were incubated with/without BAY 11-7082, IL-1β, and CTS, and Western blot was used to detect the expression of ferroptosis-related factors (P53, SLC7A11, and GPX4) and degradation-related factors (MMP13, Col2a1). The relative protein expression level was calculated in (**e**). All experiments were repeated thrice. (**f**) Effect of NF-κB overexpression on ferroptosis and degradation of osteoarthritis chondrocyte. Chondrocytes were incubated with lentivirus (20 MOI), IL-1β and CTS, then Western blot was used to detect the expression of P53, SLC7A11, GPX4, MMP13 and Col2a1. The relative protein expression level was calculated in (**g**). **P < 0.01, ***P < 0.001 compared with control; ^#^*P* < 0.05, ^##^*P* < 0.01 compared with IL-1β group; ^&^*P* < 0.05, ^&&^*P* < 0.01 compared with IL-1β + CTS + LV-control group.

The NF-κB inhibitor (BAY 11-7082) was used to inhibit the activation of the NF-κB signaling pathway to further verify the regulatory role of the NF-κB signaling pathway in chondrocyte degradation and ferroptosis. First, the optimal drug concentration of BAY 11-7082 was determined. The results of CCK8 showed that there was significant toxicity on 20 μM (*P* < 0.01; Fig. 7a). Then, 10 μM BAY 11-7082 significantly inhibited NF-κB p65 phosphorylation (*P* < 0.01; Fig. 7b). Thus, 10 μM was used in subsequent studies. Compared with the IL-1β group, the expressions of chondrocyte degradation (MMP13, Col2a1) and ferroptosis-related factors (P53, SLC7A11, GPX4) were significant differential expressions that were incubated with BAY 11-7082 (*P* < 0.01), and the expression significantly changed by 4 h CTS intervention (*P* < 0.01; Fig. 7d,e). To further verify the regulatory role of NF-κB signaling pathway on ferroptosis. Recombinant adenovirus was used to overexpress NF-κB p65. The results of qRT-PCR showed that overexpressed recombinant adenovirus at 20 and 40 MOI could significantly upregulate the expression of NF-κB p65 (*P* < 0.01 or *P* < 0.001, Fig. 7c). So, 20 MOI was used in subsequent studies. As shown in Fig. 7f,g, compared with IL-1β + CTS + LV-control group, the expression of Col2a1, SLC7A11, GPX4 were downregulated and P53, MMP13 were upregulated in IL-1β + CTS + LV-NF-kB group (*P* < 0.05, *P* < 0.01or *P* < 0.001). The overexpression of NF-κB significantly inhibited the positive effects of CTS. In summary, studies above indicated that the NF-κB p65 signaling pathway promoted IL-1β-induced chondrocytes ferroptosis and degradation by regulating the key ferroptosis-regulators P53, SLC7A11, and GPX4, thereby participating in chondrocyte ferroptosis and pathophysiological processes in OA. Mechanical stress is likely to inhibit chondrocyte ferroptosis by the NF-κB signaling pathway and alleviate OA progression.

## Discussion

OA is a common degenerative bone and joint disease. Its main pathological manifestations are cartilage degeneration, subchondral bone destruction, and osteophyte formation. Exercise therapy is safe and economical and has become the primary recommended therapy in the guidelines for treating OA. Moderate exercise can inhibit cartilage degeneration and improve chondrogenesis and cartilage regeneration^42,43^. In addition, moderate exercise has a positive effect on subchondral bone, which can accelerate subchondral bone remodeling and promote the repair of osteochondral^44,45^. Similar results were obtained in this study. The result of micro-CT showed that serious cartilage defects and decreased bone density can be observed in the articular cartilage of OA rats, meanwhile, the cartilage and subchondral bone injuries were significantly improved after treadmill exercise. Results of HE and toluidine blue staining further supported the above conclusions, the Mankin’s and OARSI score of OA cartilage was higher than that in the healthy cartilage, and the score decreased after treadmill exercise. Notably, new evidence indicated that subchondral bone is also involved in cartilage repair. There is a crosstalk between cartilage and subchondral bone, and various growth factors can be secreted during subchondral bone remodeling, thereby promoting cartilage repair^46^. Therefore, the positive effect of exercise on OA is likely by regulating the crosstalk between cartilage and subchondral bone. Therefore, it is worth to be studied deeply.

The imbalance of cartilage homeostasis is an important pathophysiological mechanism of OA. Chondrocytes regulate the anabolic/catabolic balance of the extracellular matrix to maintain the healthy structure and function of joints^47,48^. Mechanical stress is a critical environmental factor to maintain cartilage homeostasis. Chondrocytes could sense and respond the mechanical stimulation by activating a series of complex mechanical signal transduction networks, regulating the anabolic/catabolic signaling axis and inflammatory responses, and participating in OA development^9^. Thus, in this study, we found that mechanical stress could downregulate cartilage catabolic-related factors MMP13 and downregulate cartilage anabolic factor Col2a1 both in vitro and in vivo. However, mechanical stress is a double-edged sword, moderate mechanical stimulation acts as a cartilage protective factor to maintain cartilage homeostasis, however, excessive mechanical stress is one of the important risk factors for the pathogenesis of OA^49^. Studies indicated that CTS is a potent antagonist of IL-1β, could suppress IL-1β-induced chondrocyte catabolism^32^. Excessive CTS can also cause chondrocyte damage. Studies found that chondrocytes demonstrated significant fibroblastic morphology by 0.5 HZ 10% CTS, while 5% tensile maintained the chondrocyte phenotype^50,51^. In our study, we also found 0.5 HZ 5% CTS 4 h could effectively alleviate IL-1β-induced chondrocyte degeneration. But 8 h CTS intervention exacerbated chondrocyte degeneration. Therefore, there is great value in the in-depth exploration of the internal mechanical signal transduction network of chondrocytes, it is helpful to formulate exercise prescriptions scientifically and improve clinical treatment.

Chondrocyte death is closely related to the pathophysiological process of OA. A series of studies indicated that chondrocyte apoptosis, necroptosis and autophagy were identified in the cartilage tissue of OA^52,53^. Different from the above existing forms of programmed cell death, ferroptosis is discovered in recent years and is characterized by the accumulation of iron and lipid ROS^54^, but the regulatory role of ferroptosis in OA remains unclear. In addition, ferroptosis does not have the characteristics of common programmed cell death, the main morphological manifestations are mitochondrial atrophy and increased membrane density. Thus, in this study, we found a similar morphological manifestation of ferroptosis in osteoarthritic chondrocytes by TEM. Ferroptosis is regulated by a series of factors, such as GPX4, SLC7A11, and P53. GPX4 is the key regulator of ferroptosis, which can inhibit the accumulation of lipid peroxides by regulating glutathione (GSH), and inhibition of GPX4 activity is a marker of ferroptosis^12^. SLC7A11 is one of the core components of system Xc-which is an important part of the antioxidant system in cells. Inhibition of SLC7A11 could decrease GPX activity and GSH synthesis, trigger the formation of lipid ROS, and eventually lead to ferroptosis^55^. P53 has long been considered a regulator of the cancer cell cycle, senescence, and apoptosis, however, recent evidence indicated that is closely related to ferroptosis. P53 can downregulate the expression of SLC7A11 and inhibit GPX4 activity, thereby accelerating the formation of lipid ROS and the progress of ferroptosis^56^. However, whether or not P53 is involved in the process of ferroptosis in OA remains unclear. In this paper, we discovered that P53 was upregulated, meanwhile, SLC7A11 and GPX4 were downregulated in OA chondrocytes both in vitro and in vivo, thus chondrocyte ferroptosis can promote the progression of OA. To sum up, chondrocyte ferroptosis could accelerate the progression of OA, thereby leading to the degradation and degeneration of the cartilage matrix, thus targeting cell ferroptosis may be a promising direction.

Studies have discovered that that mechanical stress can maintain cartilage homeostasis by regulating programmed cell death. Zhang et al. found that moderate mechanical stress rescues chondrocytes by regulating mitochondrial function and cell apoptosis^10^. Meanwhile, Zheng et al. revealed that mechanical loading alleviates OA symptoms by regulating endoplasmic reticulum stress and cell autophagy^11^. However, whether or not the protective effect of mechanical stress is related to chondrocyte ferroptosis remains unclear. We also found that moderate mechanical stress increased the expression of GPX4 and SLC7A11, decreased the expression of P53, and Nrf2 was also activated both in vitro and in vivo. Nrf2 is the key regulator of antioxidant system which can inhibit cell ferroptosis by regulating GPX4 and ROS formation^57^. Furthermore, we found that Nrf2 was downregulated in normal chondrocytes, upregulated in IL-1β-introduced chondrocytes, and it was further upregulated by mechanical stress intervention. Therefore, mechanical stress is likely to activate Nrf2 and inhibit chondrocyte ferroptosis by regulating SLC7A11, P53, and GPX4. Currently, studies on the pathways that regulate ferroptosis are still limited.

**Figure 8:**
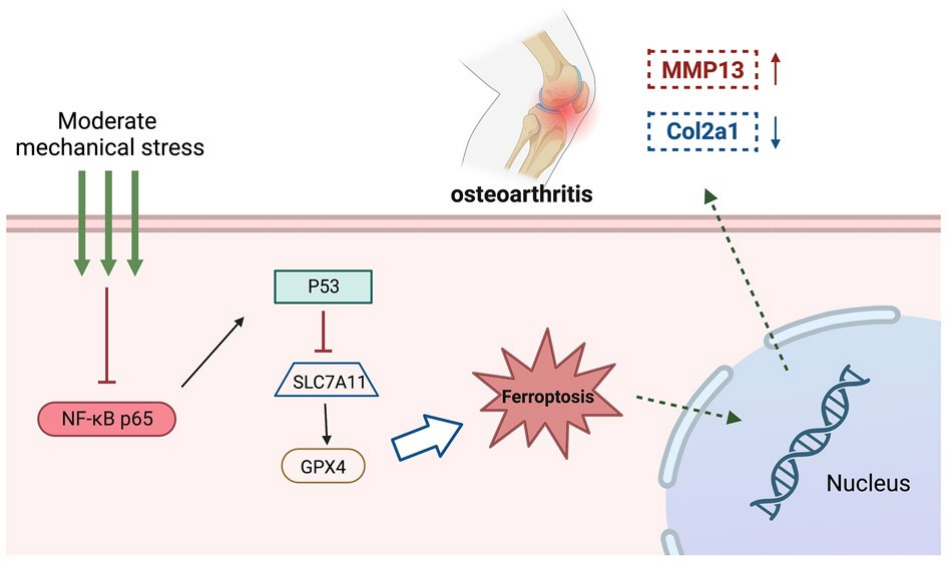
Schematic diagram of moderate mechanical stress suppresses chondrocyte ferroptosis and cartilage matrix degradation in osteoarthritis.

NF-κB p65 signaling pathway is a key signaling pathways in osteoarthritis, and studies have confirmed that NF-κB p65 signaling pathway plays a crucial role in cartilage metabolism^58^. Notably, recent evidence has found that NF-KB p65 regulates ferroptosis in a variety of diseases^39,59,60^. Xu et al. found that NF-κB p65 could suppresses endoplasmic reticulum stress-mediated endoplasmic reticulum ferroptosis in ulcerative colitis^39^. Wang et al. demonstrated that NF-κB p65 restricted ferroptosis in HepG2 and Huh7 cells by through directly binding to the core region of SLC7A11 promoter^59^. In the present study, we found that the NF-κB p65 signaling pathway was activated in OA and can be downregulated by exercise intervention. Inhibition of NF-κB signaling pathway increased the expression of Col2a1, GPX4, and SLC7A11, and decreased the expression of MMP13 and P53. Overexpression of NF-κB p65 by recombinant lentivirus can reverse the expression of these factors.Studies above showed that mechanical stress could activate Nrf2 antioxidant system, inhibit the NF-κB p65 signaling pathway, and inhibit chondrocyte ferroptosis and degeneration by regulating SLC7A11, P53, and GPX4 (Fig. 8). However, the study is not deep enough. We explored the inter molecular mechanism of chondrocytes ferroptosis only at the cellular level, lacking revalidation in NF-κB knockout mice. Furthermore, in this study, we primarily focused on cartilage and single chondrocyte ferroptosis and did not involve subchondral bone and behavior in multi-population systems. In the following studies, we will focus on the effect of mechanical stress on subchondral bone, as well as the crosstalk between cartilage and subchondral bone.

## Conclusions

We demonstrated that chondrocyte ferroptosis is closely associated with the pathogenesis and development of OA. Moderate mechanical stress could effectively inhibit cartilage degeneration and delay the progression of OA. The underlying mechanism may be that mechanical stress activates Nrf2 antioxidant system, inhibit the NF-κB p65 signaling pathway and chondrocyte ferroptosis as well as cartilage matrix degradation by regulating P53, SLC7A11, and GPX4. The current research further improved the mechanism of exercise therapy for OA and provided theoretical support for the formulation of scientific exercise prescriptions for patients with OA (Supplementary Information).

### Supplementary Information

Supplementary Information.

## Data Availability

All data and analyses supporting the findings of this study are available from the lead contact upon reasonable request.
