# Supplementary material for: Moderate mechanical stress suppresses chondrocyte ferroptosis in osteoarthritis by regulating NF-κB p65/GPX4 signaling pathway
Source: Sci Rep. 2024 Mar 1;14:5078. doi: 10.1038/s41598-024-55629-x (PMC10907644; doi:10.1038/s41598-024-55629-x)
Supplement: Supplementary file 1 — Supplementary Information. [file 41598_2024_55629_MOESM1_ESM.pdf]

All the proteins are transferred to the same PVDF membrane, before hybridisation, the PVDF membrane was cropped according to the molecular weight of the proteins to be detected.

**Figure 2c**

Col2a1

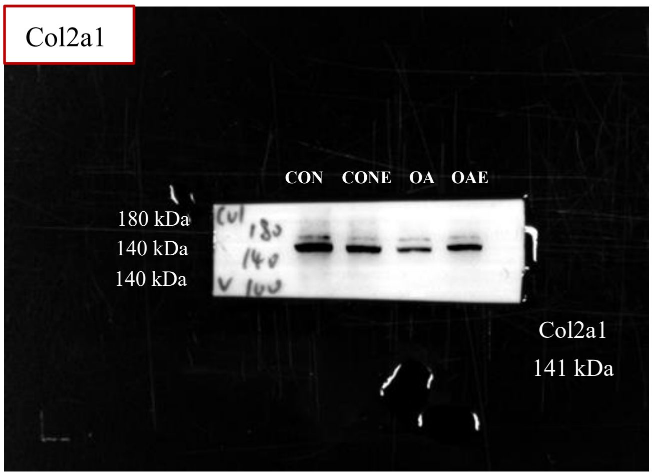


MMP13

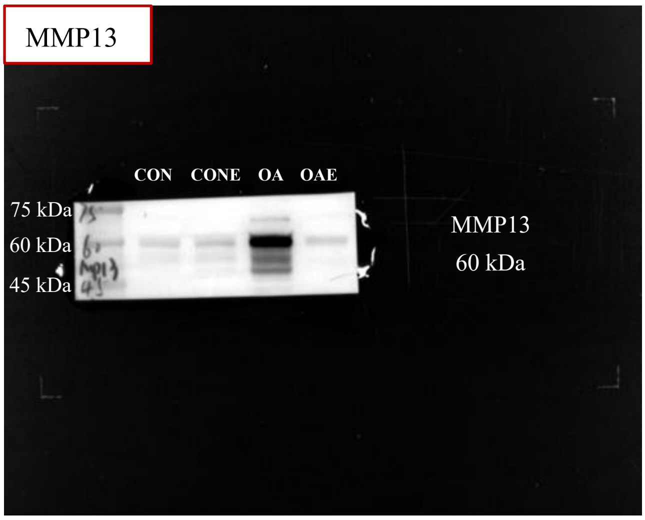


GAPDH

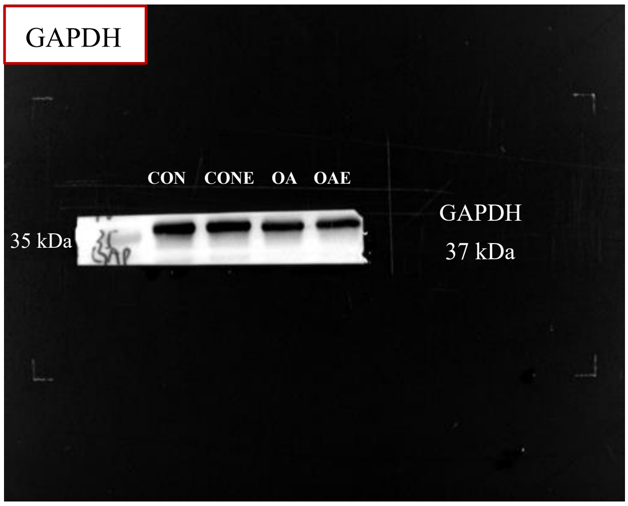

**Figure 3c**

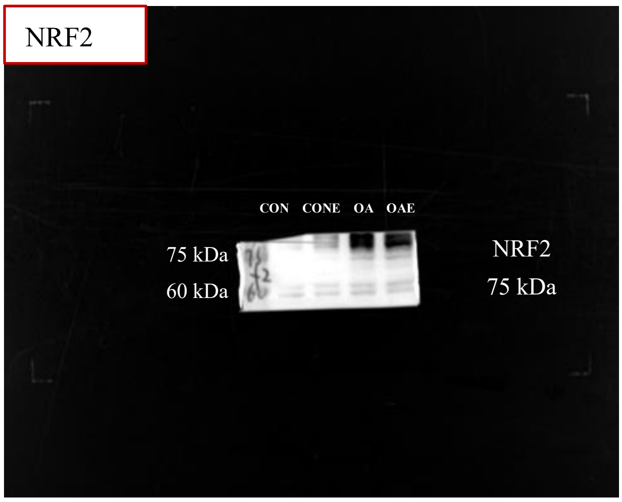
NRF2
CON CONE OA OAE
75 kDa
60 kDa
NRF2
75 kDa

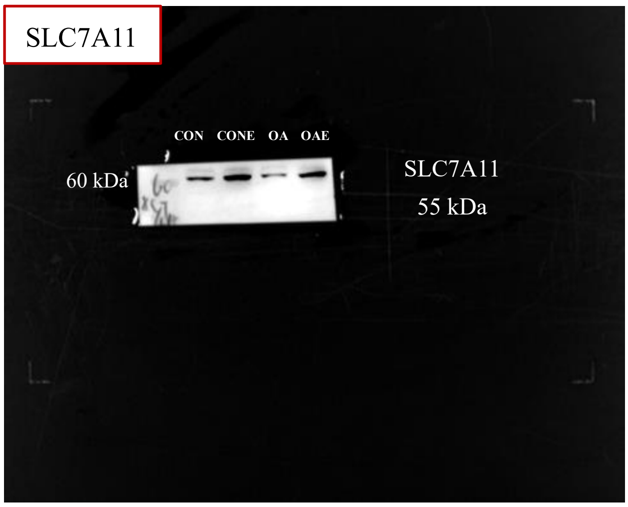
SLC7A11
CON CONE OA OAE
60 kDa
SLC7A11
55 kDa

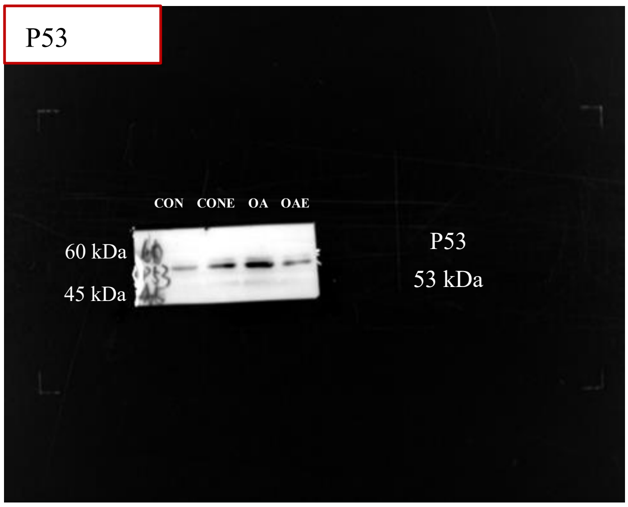
P53
CON CONE OA OAE
60 kDa
45 kDa
P53
53 kDa

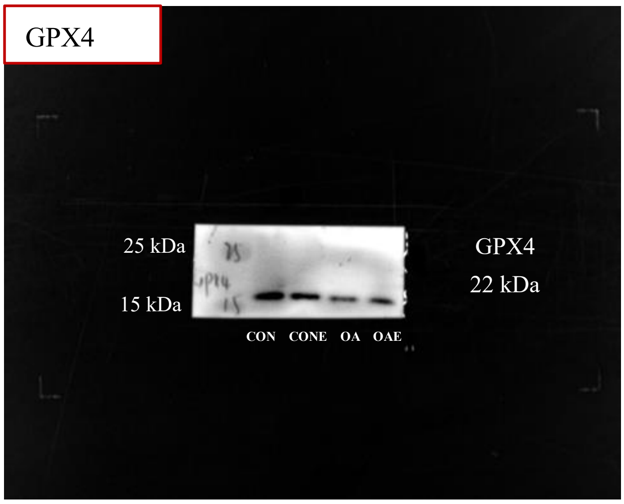
GPX4
25 kDa
15 kDa
CON CONE OA OAE
GPX4
22 kDa

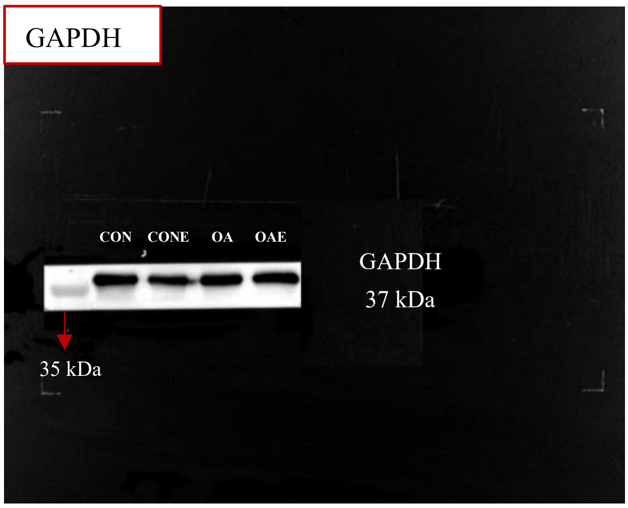
GAPDH
CON CONE OA OAE
GAPDH
37 kDa
35 kDa

**Figure 4a**

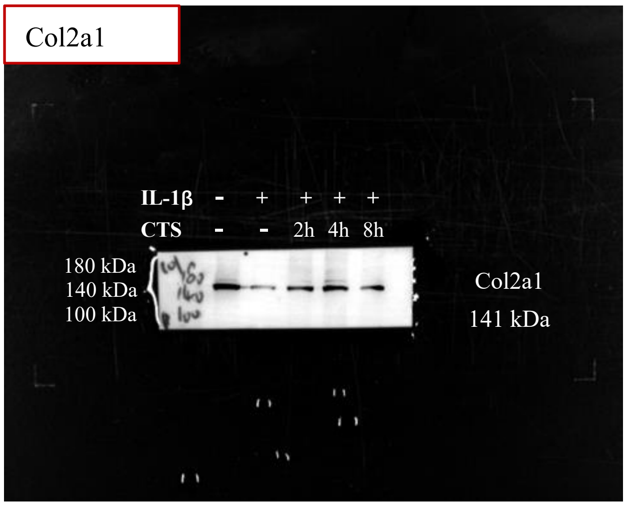
Col2a1
IL-1β - + + + +
CTS - - 2h 4h 8h
180 kDa
140 kDa
100 kDa
Col2a1
141 kDa

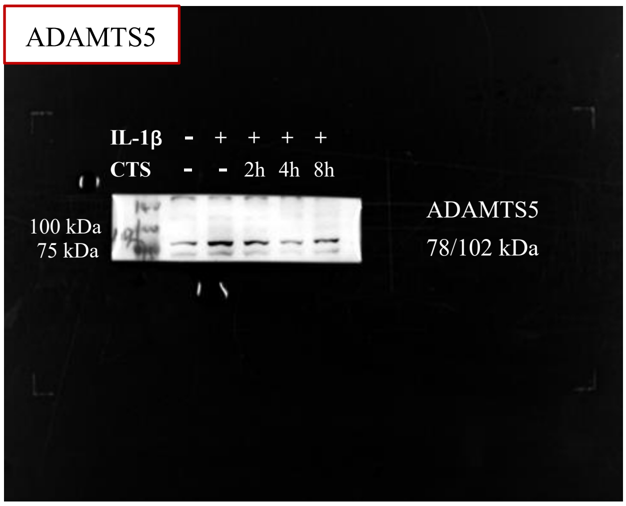
ADAMTS5
IL-1β - + + + +
CTS - - 2h 4h 8h
100 kDa
75 kDa
ADAMTS5
78/102 kDa

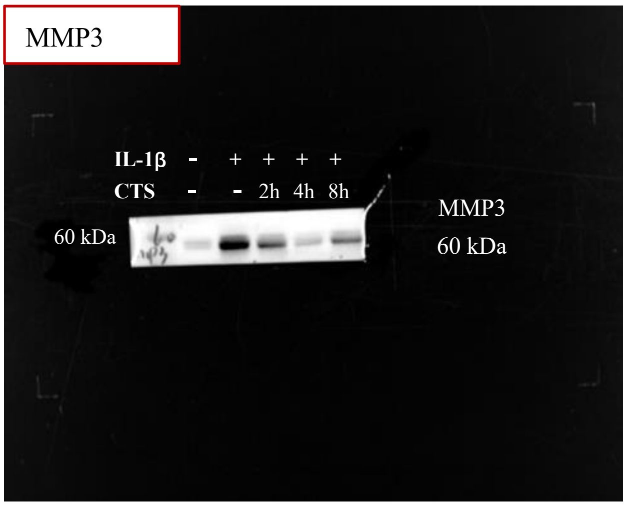
MMP3
IL-1β - + + + +
CTS - - 2h 4h 8h
60 kDa
MMP3
60 kDa

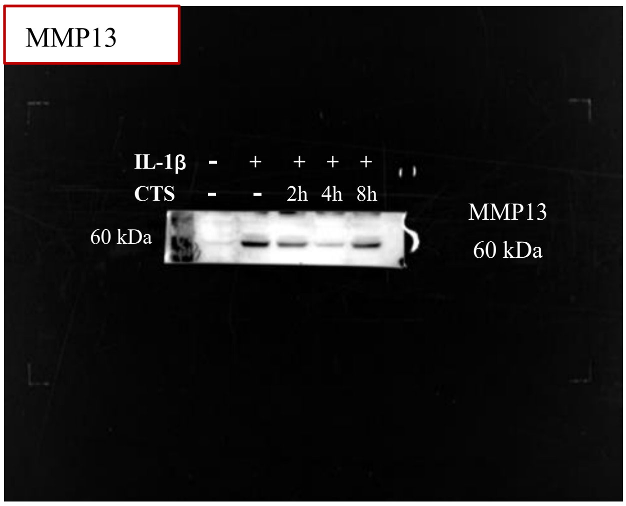
MMP13
IL-1β - + + + +
CTS - - 2h 4h 8h
60 kDa
MMP13
60 kDa

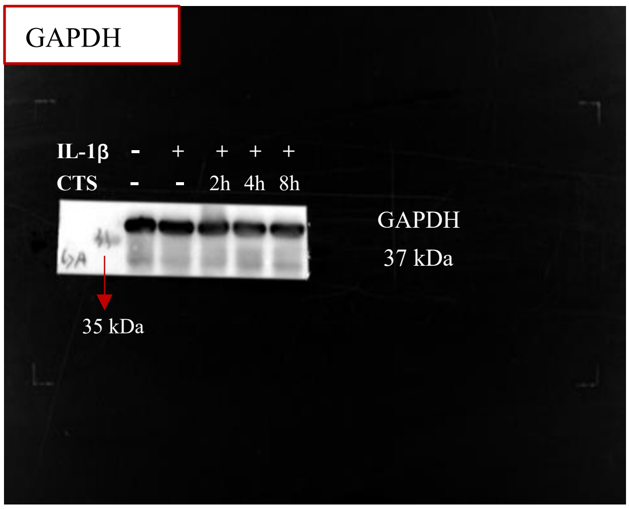
GAPDH
IL-1β - + + + +
CTS - - 2h 4h 8h
GAPDH
37 kDa
35 kDa

**Figure 5c**

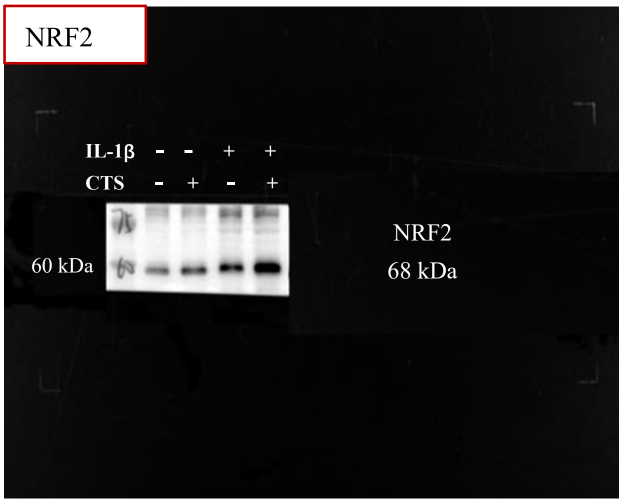
NRF2
IL-1β - - + +
CTS - + - +
60 kDa
NRF2
68 kDa

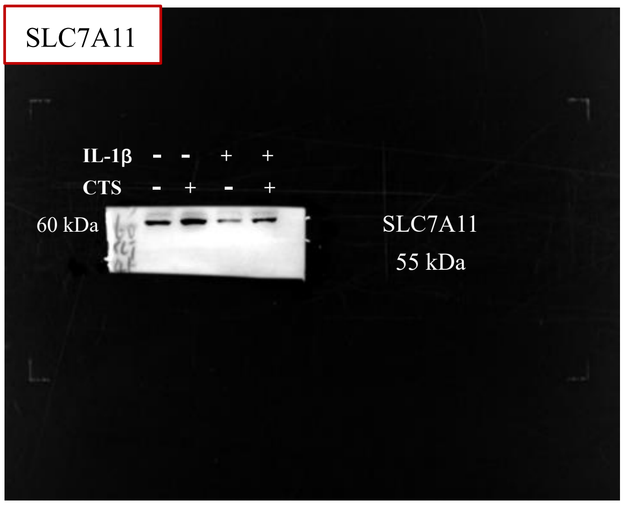
SLC7A11
IL-1β - - + +
CTS - + - +
60 kDa
SLC7A11
55 kDa

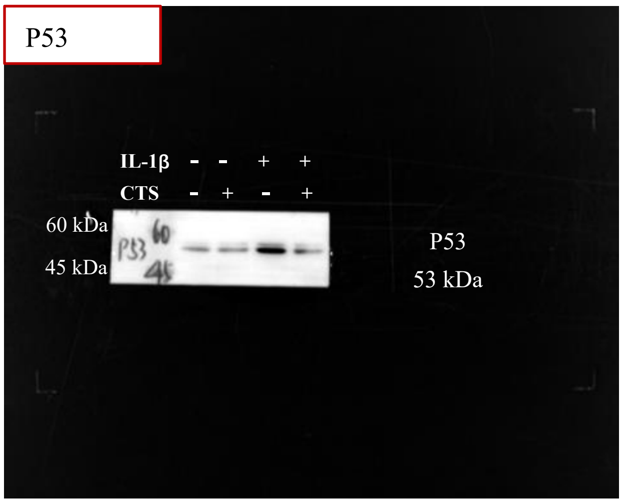
P53
IL-1β - - + +
CTS - + - +
60 kDa
45 kDa
P53
53 kDa

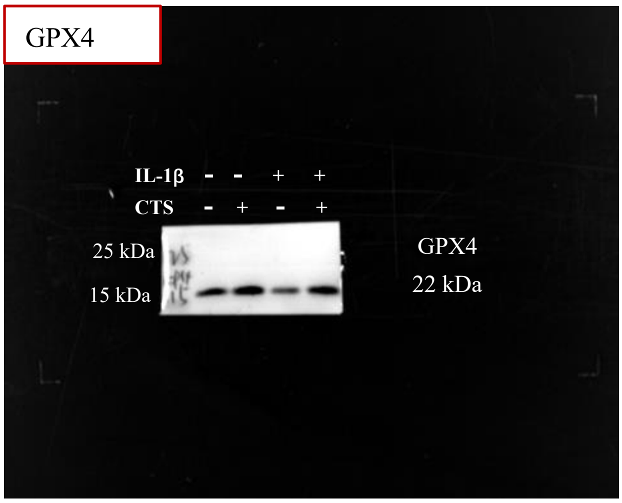
GPX4
IL-1β - - + +
CTS - + - +
25 kDa
15 kDa
GPX4
22 kDa

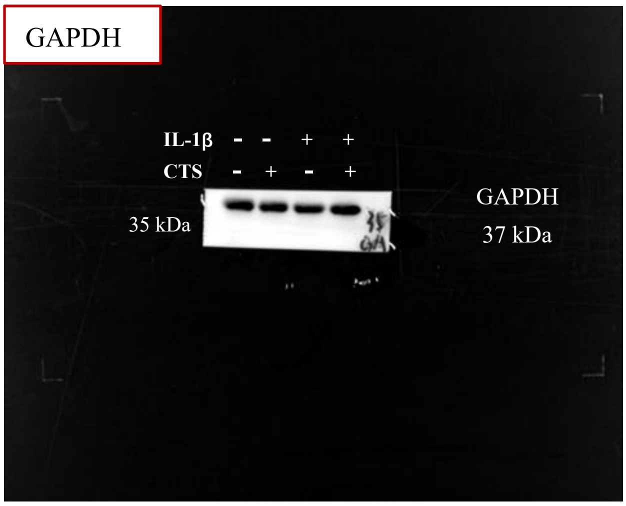
GAPDH
IL-1β - - + +
CTS - + - +
35 kDa
GAPDH
37 kDa

**Figure 6c**

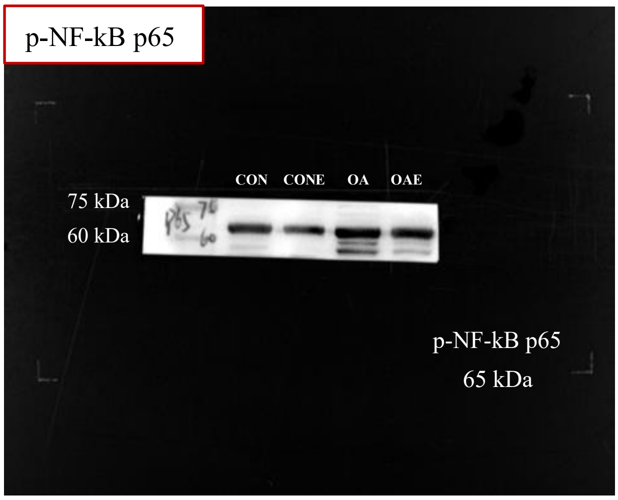
p-NF-kB p65
CON CONE OA OAE
75 kDa
60 kDa
p-NF-kB p65
65 kDa

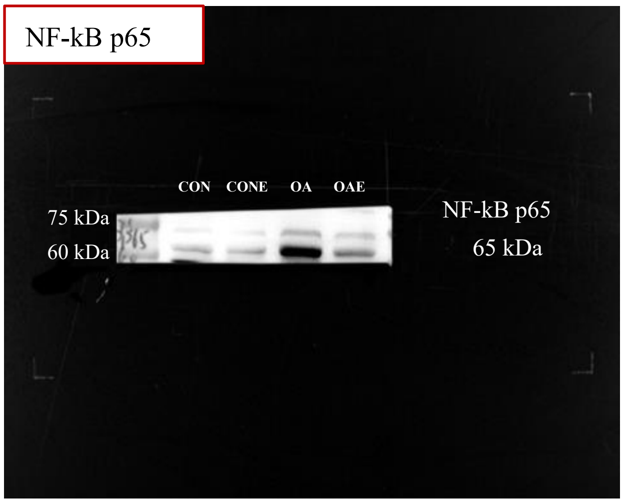
NF-kB p65
CON CONE OA OAE
75 kDa
60 kDa
NF-kB p65
65 kDa

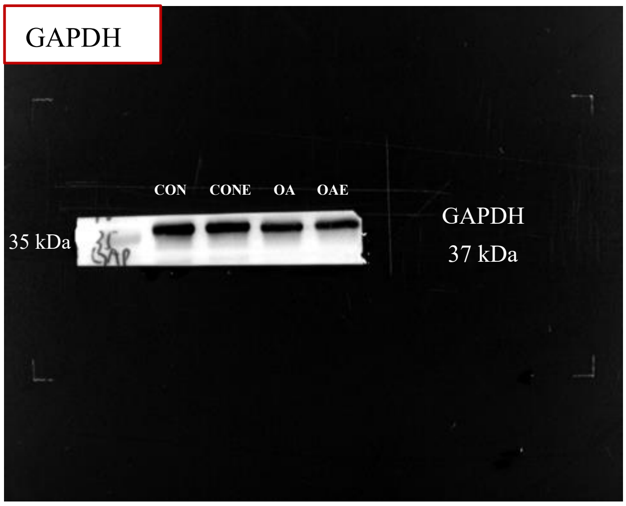
GAPDH
CON CONE OA OAE
35 kDa
GAPDH
37 kDa

**Figure 6f**

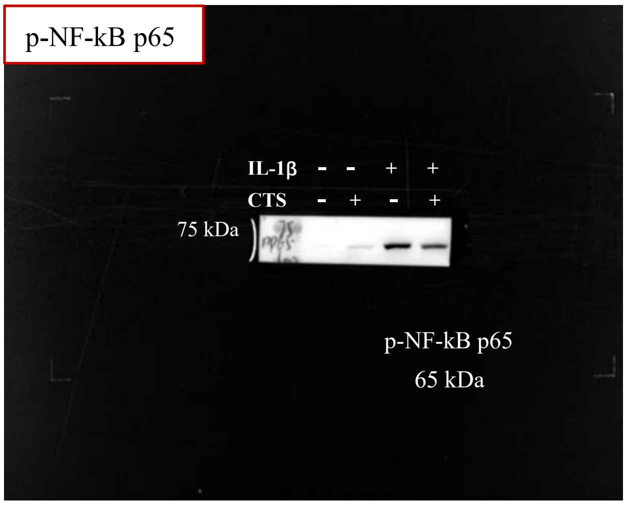
p-NF-kB p65
IL-1β - - + +
CTS - + - +
75 kDa
p-NF-kB p65
65 kDa

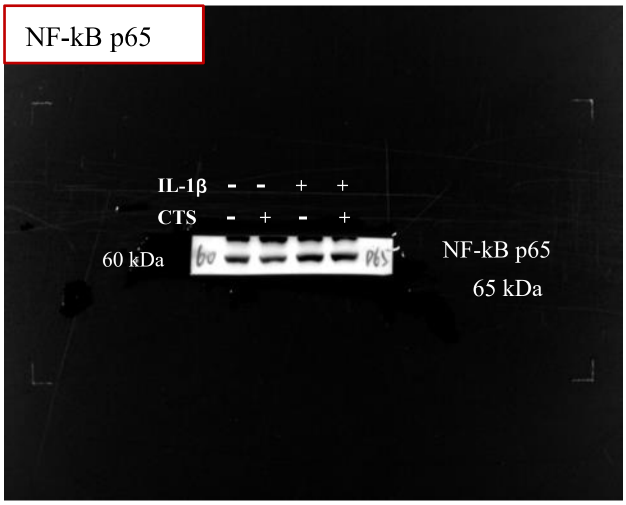
NF-kB p65
IL-1β - - + +
CTS - + - +
60 kDa
NF-kB p65
65 kDa

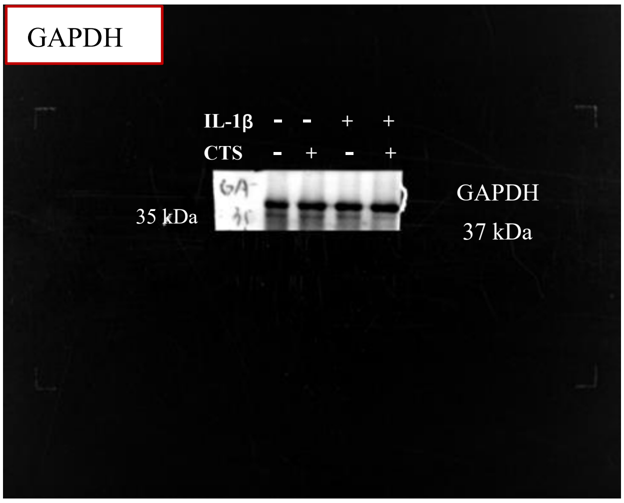
GAPDH
IL-1β - - + +
CTS - + - +
35 kDa
GAPDH
37 kDa

# Figure 7b

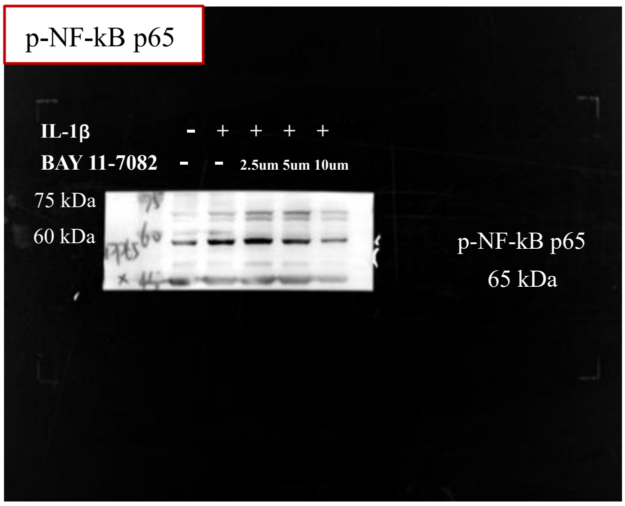
p-NF-kB p65
IL-1β - + + + +
BAY 11-7082 - - 2.5um 5um 10um
75 kDa
60 kDa
p-NF-kB p65
65 kDa

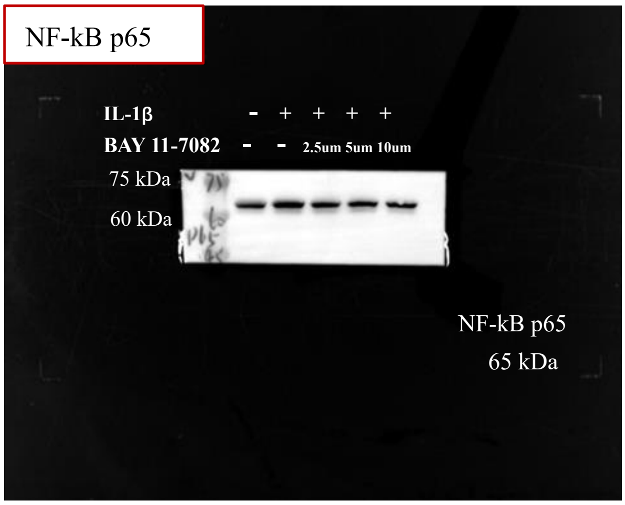
NF-kB p65
IL-1β - + + + +
BAY 11-7082 - - 2.5um 5um 10um
75 kDa
60 kDa
NF-kB p65
65 kDa

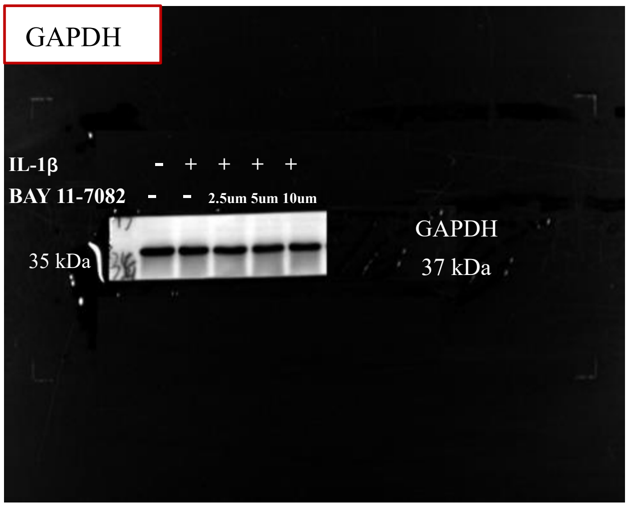
GAPDH
IL-1β - + + + +
BAY 11-7082 - - 2.5um 5um 10um
35 kDa
GAPDH
37 kDa

**Figure 7d**

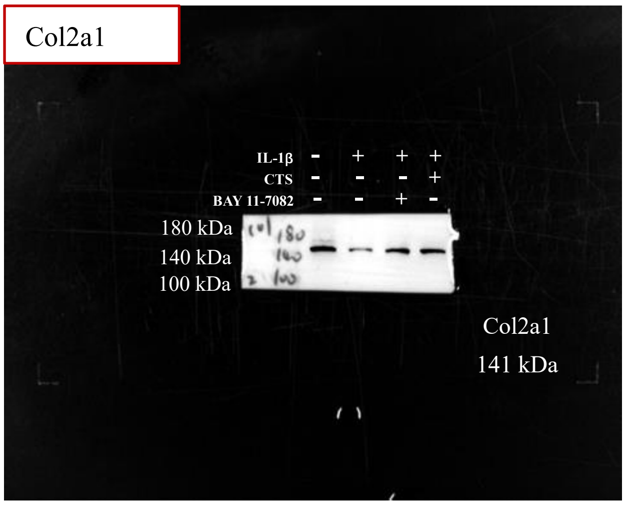
Col2a1
IL-1β - + + +
CTS - - - +
BAY 11-7082 - - + -
180 kDa
140 kDa
100 kDa
Col2a1
141 kDa

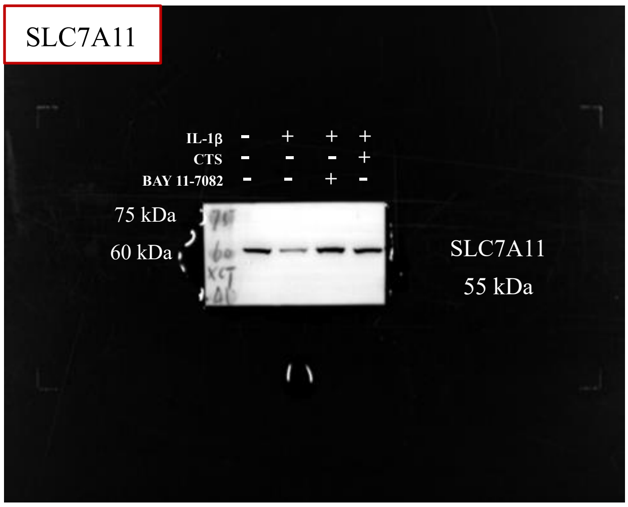
SLC7A11
IL-1β - + + +
CTS - - - +
BAY 11-7082 - - + -
75 kDa
60 kDa
SLC7A11
55 kDa

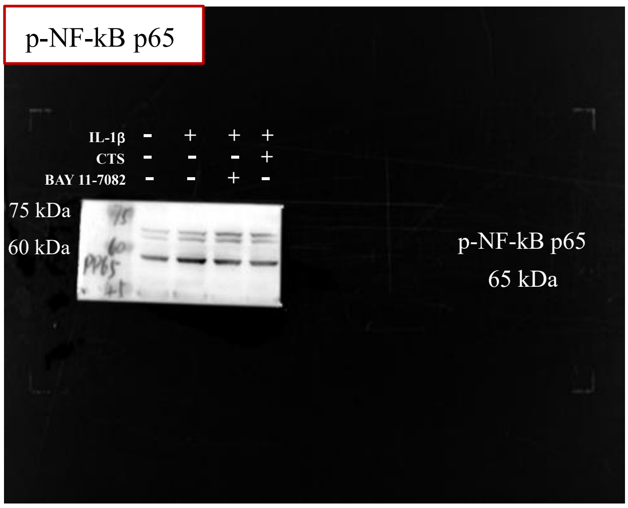
p-NF-kB p65
IL-1β - + + +
CTS - - - +
BAY 11-7082 - - + -
75 kDa
60 kDa
p-NF-kB p65
65 kDa

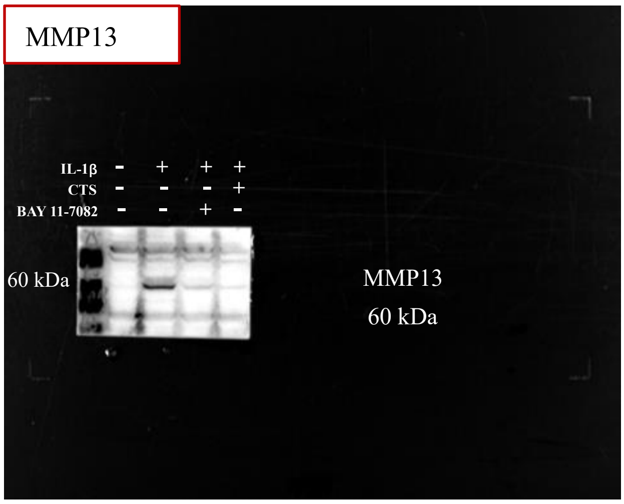
MMP13
IL-1β - + + +
CTS - - - +
BAY 11-7082 - - + -
60 kDa
MMP13
60 kDa

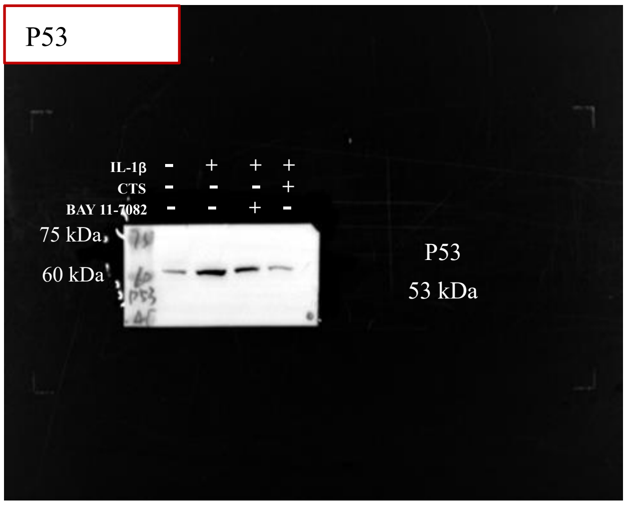
P53
IL-1β - + + +
CTS - - - +
BAY 11-7082 - - + -
75 kDa
60 kDa
P53
53 kDa

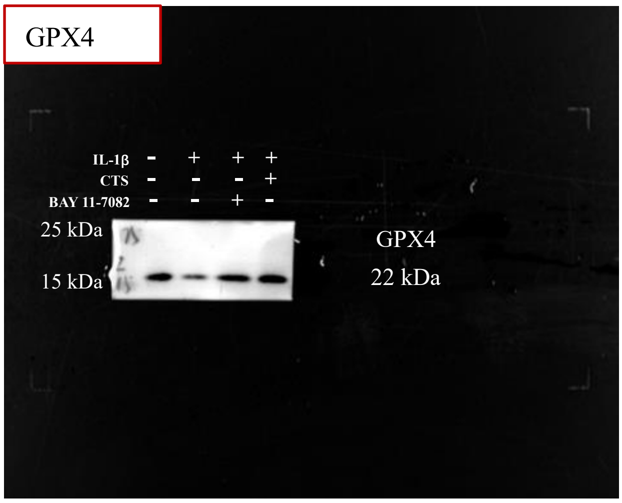
GPX4
IL-1β - + + +
CTS - - - +
BAY 11-7082 - - + -
25 kDa
15 kDa
GPX4
22 kDa

GAPDH

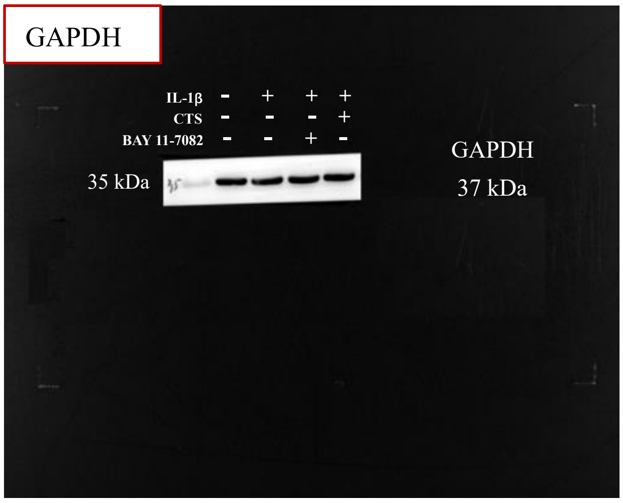


**Figure 7f**

Col2a1

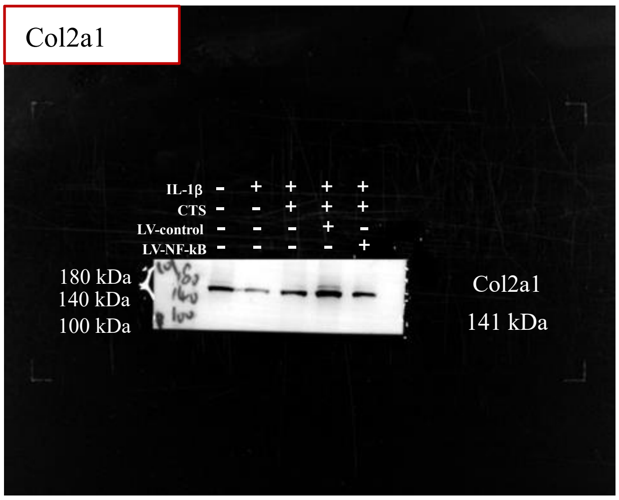


SLC7A11

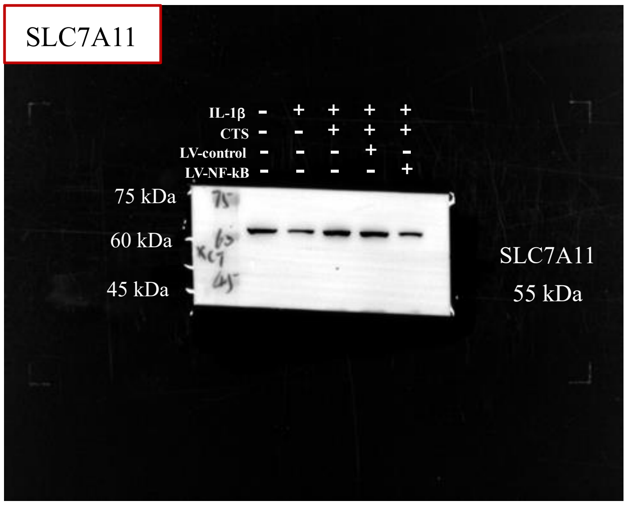

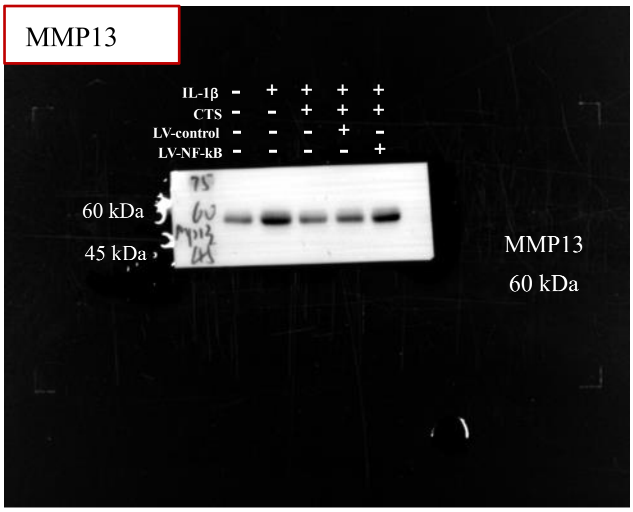
MMP13
IL-1β - + + + +
CTS - - + + +
LV-control - - - + -
LV-NF-kB - - - - +
60 kDa
45 kDa
MMP13
60 kDa

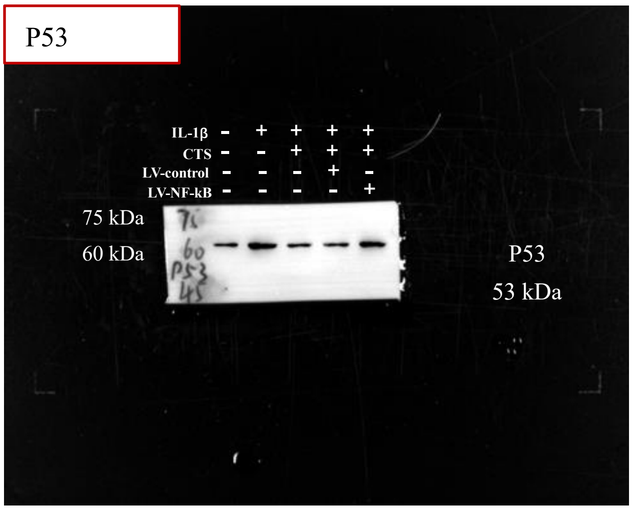
P53
IL-1β - + + + +
CTS - - + + +
LV-control - - - + -
LV-NF-kB - - - - +
75 kDa
60 kDa
P53
53 kDa

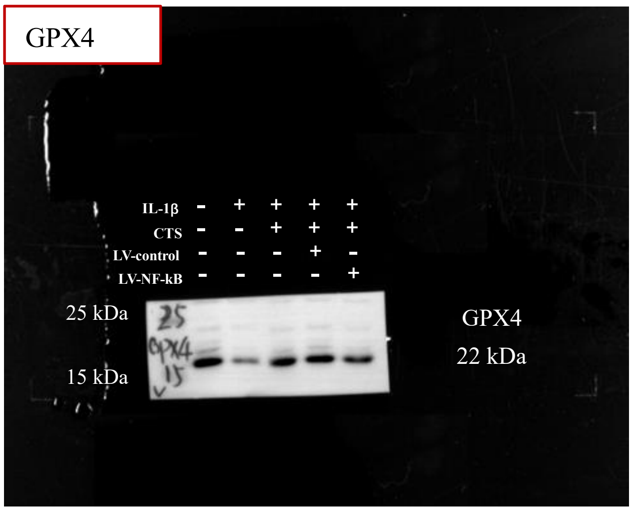
GPX4
IL-1β - + + + +
CTS - - + + +
LV-control - - - + -
LV-NF-kB - - - - +
25 kDa
15 kDa
GPX4
22 kDa

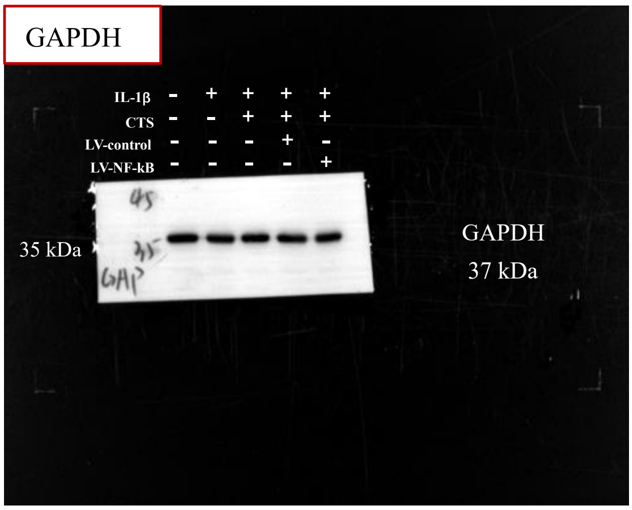
GAPDH
IL-1β - + + + +
CTS - - + + +
LV-control - - - + -
LV-NF-kB - - - - +
35 kDa
GAPDH
37 kDa
